# Seven-day versus fourteen-day low-dose primaquine for *Plasmodium vivax* malaria in India: an open-label, non-inferiority, randomised, controlled trial

**DOI:** 10.1016/j.lansea.2026.100849

**Published:** 2026-08-25

**Authors:** Rajendra Kumar Baharia, Sauman Singh-Phulgenda, Syed Mohammad Naser, Apoorv Gupta, Rutuja Chhajed, Mohammad Sharif Hossain, Karmveer Yadav, Reena Verma, Salil Kumar Pal, Parinita Halder Ranjit, Bhavin Solanki, Kamlesh Jayantilal Upadhyay, Leena Dabhi, Rajesh Sharma, Subrata Roy Chowdhury, Amitava Chakravorty, Megha Rajasekhar, Grant Lee, Hellen Mnjala, Souvik Ghosh, Sunitaben S. Parmar, Debjani Sengupta, Anup Anvikar, Philippe J. Guerin, Ric N. Price, Manju Rahi, Praveen K. Bharti, Kamala Thriemer, Nitika Nitika, Robert J. Commons

**Affiliations:** a ICMR- National Institute of Malaria Research Field Unit, Nadiad, Gujarat, India; b Academy of Scientific and Innovative Research (AcSIR), Ghaziabad, India; c WorldWide Antimalarial Resistance Network (WWARN), Oxford, UK; d Infectious Diseases Data Observatory (IDDO), Oxford, UK; e Centre for Tropical Medicine and Global Health, Nuffield Department of Clinical Medicine, University of Oxford, Oxford, UK; f Calcutta National Medical College, Kolkata, West Bengal, India; g ICMR-National Institute of Malaria Research, Sector 8, Dwarka, Delhi, 110077, India; h International Centre for Diarrhoeal Disease Research, Bangladesh (icddr,b), Dhaka, Bangladesh; i Ahmedabad Municipal Corporation, Ahmedabad, Gujarat, India; j BJ Medical College, Ahmedabad, Gujarat, India; k Kolkata Municipal Corporation, Kolkata, West Bengal, India; l Centre for Epidemiology and Biostatistics, Melbourne School of Population and Global Health, The University of Melbourne, Melbourne, VIC, Australia; m Global and Tropical Health Division, Menzies School of Health Research and Charles Darwin University, Darwin, NT, Australia; n WorldWide Antimalarial Resistance Network (WWARN), Asia-Pacific Regional Centre, Melbourne, VIC, Australia; o ICMR-Vector Control Research Centre, Puducherry, India; p General and Subspecialty Medicine, Grampians Health-Ballarat, Ballarat, VIC, Australia

**Keywords:** *Plasmodium vivax*, Malaria, Primaquine, Glucose-6-phosphate dehydrogenase deficiency, Multicentre randomised trial

## Abstract

**Background:**

India has a high burden of *Plasmodium vivax* malaria. Current malaria treatment guidelines recommend a total primaquine dose of 3·5 mg/kg administered over 14 days to prevent relapse in *P. vivax* infection, however, adherence is generally poor, impacting effectiveness. We aimed to determine the efficacy and safety of the same total dose of 3·5 mg/kg administered over 7 days in India.

**Methods:**

We conducted an open label, non-inferiority, randomised controlled trial at two sites in India. Febrile patients older than 16 years with microscopically confirmed *P. vivax* mono-infection and normal (≥30%) G6PD activity were eligible for enrolment. Patients were randomly assigned (1:1) using block randomisation, stratified by site, to either 3·5 mg/kg primaquine over 7 days or 3·5 mg/kg primaquine over 14 days, which is the current standard of care. Primaquine was administered in combination with chloroquine as per national treatment guidelines. The primary endpoint was the cumulative incidence of symptomatic recurrent microscopy-confirmed *P. vivax* parasitaemia within 6 months. Efficacy analyses were conducted on the intention-to-treat population. The study was registered with the Clinical Trials Registry of India (CTRI/2022/12/048283).

**Findings:**

Between September 2023 and October 2024, a total of 400 patients were enrolled (201 randomised to the 7-day group and 199 to the 14-day group). By 180 days of follow up, there were a total of four *P. vivax* recurrences, with two in each arm. The cumulative incidence of symptomatic parasitaemia by day 180 was 1·1% (95% confidence interval [CI] 0·0–4·1) in the 7-day primaquine group and 1·1% (95% CI 0·0–4·4) in the 14-day primaquine group. Sixteen patients (8%) treated with 7-day primaquine missed a primaquine dose compared with 40 patients (20%) treated with 14-day primaquine. There were 35 adverse events reported in each group, 11 of which were reported to be drug-related in the 7-day group compared with 13 in the 14-day group. Gastrointestinal side effects at day 7 were rare in both the 7-day (0·5% [1/183]) and the 14-day group (1·1% [2/180]). None of the patients had a haemoglobin fall from day 0 to days 1–14 by more than 5 g/dL or required blood transfusion.

**Interpretation:**

Compared with a 14-day primaquine regimen, a 7-day regimen of 3·5 mg/kg was well-tolerated and non-inferior within the observed 180 days of follow up. Adherence was greater among patients in the 7-day regimen. Although the overall number of recurrences was lower than anticipated, limiting the precision of the estimates, these findings are consistent with and support the current WHO recommendation for a 7-day primaquine regimen. Future studies with longer follow up could provide additional evidence for efficacy of this regimen against late *P. vivax* relapses.

**Funding:**

Infectious Diseases Data Observatory (University of Oxford) through a grant from the Bill & Melinda Gates Foundation (INV-004713).

**Research in context**

**Evidence before this study**

India currently recommends a 14-day primaquine regimen of 0·25 mg/kg/day (3·5 mg/kg total dose) to prevent *Plasmodium vivax* malaria relapses. WHO guidelines were updated in 2022 to recommend the same total dose administered over 7 days. We searched for randomised controlled trials comparing 7-day and 14-day primaquine regimens for uncomplicated *P. vivax* malaria in India published between January 1, 2000, and July 3, 2026 in any language using the search terms ‘vivax’, ‘primaquine’ and ‘India’ in MEDLINE, Embase, Scopus, Web of Science and the Cochrane Database of Systematic reviews. We identified two studies, however, only one study used the standard primaquine formulation that is currently available. This study was conducted in a single centre between 2001 and 2004 and compared 7-day and 14-day primaquine at a total dose of 3·5 mg/kg. The risk of recurrence within 6 months in the 14-day arm was 8·1% compared with 10·1% in the 7-day arm. Both regimens were found to be safe and well tolerated.

**Added value of this study**

Our open-label, non-inferiority, randomised controlled trial conducted in two sites in India assessed the efficacy and safety of primaquine administered over 7 days (0·5 mg/kg/day) in comparison to the same total dose administered over 14 days (0·25 mg/kg/day) to prevent *P. vivax* recurrences among patients (≥16 years) with uncomplicated *P. vivax* malaria. Both treatments resulted in a low risk of recurrence within 180 days and were tolerated well among patients with normal (≥30%) G6PD activity. Adherence was lower among patients treated with 14 days of primaquine compared to 7 days of primaquine. Given the potential for relapse beyond the 180-day follow-up period, studies with longer follow up could provide additional data to establish the efficacy of this regimen post 6 months.

**Implications of all the available evidence**

Primaquine regimens with a total dose of 3·5 mg/kg for anti-relapse treatment of *P. vivax* are safe and demonstrate good efficacy over 6 months when given over 7 days or 14 days to patients with normal G6PD activity. However, adherence to treatment is better in the shorter regimen and could enhance effectiveness. This evidence supports the WHO recommendation for the 7-day primaquine regimen for anti-relapse treatment of *P. vivax* infections in India. Given the potential for relapse beyond the 180-day follow-up period, studies with longer follow up could provide additional data to establish the efficacy of this regimen post 6 months.

## Introduction

*Plasmodium vivax* malaria poses a major challenge to malaria control and elimination efforts globally, with the greatest burden in Asia, the Horn of Africa and Latin America.^1^ Unlike *Plasmodium falciparum*, *P. vivax* forms dormant liver hypnozoites that can cause relapses within weeks to months after the primary infection, contributing to morbidity and onward transmission.^2^ India carries one of the largest burdens of *P. vivax* worldwide, and relapses are one of the key barriers to achieving the country's malaria elimination targets.^1^

To reduce the risk of relapses, treatment of both the acute blood stage and the dormant liver stage of the parasite are required, which is referred to as radical cure. The most widely used anti-relapse treatment is primaquine. In some locations the low-dose primaquine regimen of 0·25 mg/kg/day administered over 14 days, corresponding to a total dose 3·5 mg/kg, is considered to be efficacious when taken in its entirety.^3^^,^^4^ However, adherence to the 14-day regimen is poor, undermining its effectiveness.^5^, ^6^, ^7^, ^8^

To address this, the World Health Organization (WHO) updated its guidelines in 2022 to recommend an alternative regimen of 0·5 mg/kg/day for 7 days.^9^ This regimen has the same total dose of 3·5 mg/kg but the shorter course has the potential to improve adherence and therefore effectiveness.

In 2024, WHO recommended an increase in the total dose of primaquine from 3·5 mg/kg to 7 mg/kg.^10^ This recommendation was based on an individual patient data pooled meta-analysis that provided data on primaquine regimens of different doses and durations.^11^ More recent studies have confirmed the efficacy and tolerability of high-dose regimens, including trials in the Asia–Pacific region.^11^, ^12^, ^13^, ^14^, ^15^, ^16^ However, in an individual patient data meta-analysis of studies from South Asia there was only a small absolute benefit of increasing the total dose of primaquine from 3·5 mg/kg to 7 mg/kg.^5^

There are no contemporary data on different primaquine regimens from India to inform policy and programme implementation. While shorter treatment options may increase effectiveness, the higher daily doses required may also increase the risk of haemolysis, particularly among individuals with undiagnosed glucose-6-phosphate dehydrogenase (G6PD) deficiency. Reliable quantitative G6PD testing is therefore essential to guide safe dosing and enable wider adoption of these more effective radical cure strategies.^17^

The aim of our multicentre, open-label, non-inferiority, randomised trial was to compare the efficacy, safety, and tolerability of a 7-day primaquine low-dose regimen (0·5 mg/kg/day) with the current standard of care, 14-day low-dose regimen (0·25 mg/kg/day), when co-administered with chloroquine, among patients with microscopically confirmed *P. vivax* malaria and normal G6PD activity.

## Methods

This was an open-label, non-inferiority, randomised controlled trial conducted at two sites in India (Calcutta National Medical College (CNMC), Kolkata, and Ahmedabad, Gujarat). The trial evaluated the efficacy, safety and tolerability of the 7-day regimen (0·5 mg/kg per day) compared to the 14-day regimen (0·25 mg/kg per day) for the radical cure of *P*. *vivax* malaria.

The study was a joint project, conducted by the Indian Council of Medical Research, National Institute for Malaria Research (ICMR–NIMR) in collaboration with the Infectious Diseases Data Observatory (IDDO), University of Oxford and the Menzies School of Health Research. The protocol was approved by the institutional ethics committees ICMR-NIMR as well as participating sites and registered with the Clinical Trials Registry of India (CTRI/2022/12/048283) and has been published previously.^18^ Oversight of trial conduct and safety was provided by an independent Data and Safety Monitoring Board (DSMB). The study is reported according to CONSORT guidelines using the extension for non-inferiority trials (Supplementary File 3, pp 2–8). Public involvement in the design of the trial was primarily achieved through ongoing engagement with the National Malaria Control Programme, which consistently emphasised the need to generate local evidence on the effectiveness of novel primaquine regimens, and our trial was developed in direct response to these priorities.

Patients older than 16 years with fever or a history of fever within the previous 48 h presenting to participating health centres were screened for malaria by rapid diagnostic test, followed by microscopy. Those with microscopically confirmed *P*. *vivax* mono-infection, with haemoglobin of 8·0 g/dL or more, and normal quantitative G6PD activity (≥30% of the adjusted male median by spectrophotometry) were eligible for enrolment.

Exclusion criteria included participants with mixed *Plasmodium* species infection, severe malaria as defined by WHO criteria, pregnancy or breastfeeding, bodyweight less than 40 kg (due to a high primaquine mg/kg dose below this), or receipt of any antimalarial treatment within 30 days prior to enrolment. Patients with known hypersensitivity to chloroquine or primaquine, a history of severe haemolysis, methaemoglobinaemia, blood transfusion within the past 90 days, or another serious comorbidity or illness were also excluded from the study. Full eligibility criteria, which are consistent with a recent non-inferiority trial comparing tafenoquine with 14-day low-dose primaquine,^19^ are provided in the trial protocol (Supplementary File 1).

Eligible patients were referred from community health centres linked to the municipal health networks in Ahmedabad and Kolkata or directly recruited from outpatient departments at associated hospitals. Sites included four community health centres (Vatva, Vastral, Sarkhej, Rakhiyal) and L.G. General Hospital under the Ahmedabad Municipal Corporation, and twenty-two centres under the Kolkata Municipal Corporation Supplementary File 3, p 9.

Patients were randomly assigned in a 1:1 ratio to receive either primaquine 0·5 mg/kg per day for 7 days or primaquine 0·25 mg/kg per day for 14 days. Randomisation was performed centrally via a REDCap electronic data capture system. A computer-generated schedule using block randomisation (block size 10), stratified by study site, was prepared by an independent statistician not involved in data management or analysis. Allocation was implemented automatically in REDCap once eligibility was confirmed, ensuring concealment until assignment. The trial was open-label so no masking of study personnel or patients was undertaken.

### Procedures

All eligible patients presenting with fever underwent malaria rapid diagnostic testing and microscopy for parasite identification and quantification. Additional investigations included dengue NS1 antigen testing, a urine pregnancy test if appropriate, and quantitative G6PD testing by ultraviolet spectrophotometry. Patients with microscopy-confirmed *P. vivax* infection received their first dose of chloroquine on the same day.

On the next day (day 0), when spectrophotometry results were available, patients with G6PD activity ≥30% and ≥8·0 g/dL haemoglobin (measured by HemoCue™) were enrolled into the study. Demographic data, medical and treatment history and physical examination findings were recorded. Laboratory tests included complete blood counts and liver function tests.

The patients were assigned randomly to one of the two treatment arms:

• 7-day arm: primaquine 0·5 mg/kg per day for 7 consecutive days plus chloroquine (25 mg base/kg over 3 days). Dosed as two 15 mg tablets for participants 40–70 kg and three 15 mg tablets for participants >70 kg.
• 14-day arm: primaquine 0·25 mg/kg per day for 14 consecutive days plus chloroquine (25 mg base/kg over 3 days). Dosed as one 15 mg tablet for participants 40–70 kg and one and a half 15 mg tablets for participants >70 kg.

The 14-day primaquine arm is consistent with a recent trial comparing novel anti-relapse regimens with 14-day low-dose primaquine.^19^ All chloroquine doses and the first three doses of primaquine were administered under direct observation. Subsequent primaquine doses were supervised on alternate days, with the next day's dose provided to patients for self-administration at home. Study staff confirmed adherence at each follow-up visit. All primaquine and chloroquine tablets used in the study were obtained from a single batch manufactured by Ipca Laboratories Ltd. (India) and supplied through the National Centre for Vector Borne Disease Control (NCVBDC).

Patients were reviewed on days 1, 2, 7, 14, 21, and 28, and monthly thereafter until the 6th month. At each visit, a malaria symptom questionnaire, clinical examination, visual assessment of urine, haemoglobin measurement (HemoCue™) and blood smears for microscopy were undertaken. Adverse events were recorded throughout follow-up. The site medical officer and Principal Investigator assessed the relationship between treatment and adverse events, and this was further reviewed by three additional medically trained study investigators. Study staff contacted patients who missed scheduled follow-up visits and encouraged them to return for evaluation. Patients were instructed to report to the study site if they experienced any symptoms. The schedule of screening, enrolment, interventions, and assessments is presented in Supplementary File 1.

Data were recorded on paper-based case report forms at each site and subsequently entered into the electronic case report form in REDCap, hosted on a secure local server at ICMR–NIMR, New Delhi. Each entry was reviewed by a site data entry operator and verified by a dedicated data manager at NIMR. Routine data quality checks were performed, and discrepancies were resolved with sites. Hybrid site monitoring was undertaken by a joint team from IDDO, the Menzies School of Health Research, and ICMR–NIMR to ensure protocol compliance, data accuracy, and overall trial integrity. Quality control of microscopy was undertaken centrally at NIMR in New Delhi for both sites.

### Outcomes

The primary outcome was the cumulative incidence of symptomatic microscopically-confirmed recurrent *P*. *vivax* parasitaemia within 6 months after enrolment. Secondary outcomes included the cumulative incidence of any recurrence (symptomatic or asymptomatic *P. vivax* parasitaemia) within 6 months, the number of adverse events within 28 days of initial treatment, the number of serious adverse events within 6 months of initial treatment, the proportion of patients with severe anaemia (haemoglobin <8 g/dL) and/or the risk for blood transfusion on days 1–14, the risk of a greater than 25% fall in haemoglobin from day 0 to days 1–14, the proportion of patients with an acute drop in Hb of >5 g/dL from day 0 to days 1–14, the proportion of patients vomiting primaquine within 1 h of administration and the proportion of patients with a composite outcome of any of abdominal pain, diarrhoea, nausea, vomiting or anorexia on each of days 2, 7, and 14.

All microscopy and laboratory assessments followed standard operating procedures and microscopy was validated by ICMR–NIMR. Complete blood count, liver function tests, and quantitative G6PD measurements by spectrophotometry were performed at a central reference laboratory serving both study sites to ensure standardisation and quality control.

### Statistical analysis

A non-inferiority approach was used as previous studies had not demonstrated an impact on the risk of recurrence based on the duration of primaquine treatment.^11^ Due to resource and financial constraints, the maximum number of patients was limited to 200 patients in each arm. We anticipated a 30% loss to follow up at day 180, although the actual loss to follow up was significantly lower (7%). The anticipated sample size of 134 patients per arm (268 total) would have provided 68% power based on an assumed cumulative incidence of recurrence of 13% with 14-day primaquine,^20^ a 10% non-inferiority margin and a one-sided significance level of 0·025. All analyses were conducted in accordance with the Statistical Analysis Plan (Supplementary File 2). Efficacy analyses were based on the intention-to-treat population, defined as all randomised patients according to their assigned treatment groups, regardless of adherence to the protocol or treatment received. The per-protocol population included patients without major protocol deviations, and safety analyses included all patients who received any study drug.

Continuous variables were described by mean and standard deviation or median and interquartile range depending on their distribution. The efficacy endpoints were analysed using Kaplan–Meier survival analysis with the end time the first of the day of last follow up, *P. falciparum* parasitaemia, day 180 or the day of the event *(P. vivax* parasitaemia). A Cox regression model was used to estimate the hazard ratio of recurrences with the 7-day primaquine arm compared with the 14-day arm. A sensitivity analysis was undertaken stratifying by study site. Safety and tolerability outcomes were summarised descriptively by treatment groups.

Analyses were conducted using Stata version 19 (StataCorp, College Station, TX, USA). Full details of censoring rules, missing-data handling and analyses are provided in the Statistical Analysis Plan (Supplementary File 2).

### Ethics statement

ICMR- National Institute of Malaria Research obtained approval from the Institutional Ethics committee (IEC) [NIMR/ECM/2022/Nov/N-1] with relevant documents after scientific advisory committee (SAC) approval. Ethics approval was also obtained from both site ethics committees [Ahmedabad: EC/Approval/85/2022, Kolkata: EC/CNMC/2022/13]. The proposal was also submitted for Health Ministry Screening Committee's (HMSC) approval. Written informed consent was obtained from all patients before screening. For patients younger than 18 years, written assent was obtained along with consent from a parent or guardian. Trial conduct and safety oversight were provided by an independent Data and Safety Monitoring Board (DSMB).

### Role of the funding source

The funder had no role in study design, data collection, data analysis, data interpretation, or writing of the report. The corresponding author had full access to all study data and had final responsibility for the decision to submit the manuscript for publication.

## Results

Between September 11, 2023, and October 15, 2024, a total of 6000 patients were screened for eligibility, of whom 400 patients (6·7%) were enrolled and randomised. Among them, 201 were assigned to the 7-day group and 199 to the 14-day group (Fig. 1 and Supplementary File 3 pp 12–13).

**Fig. 1:**
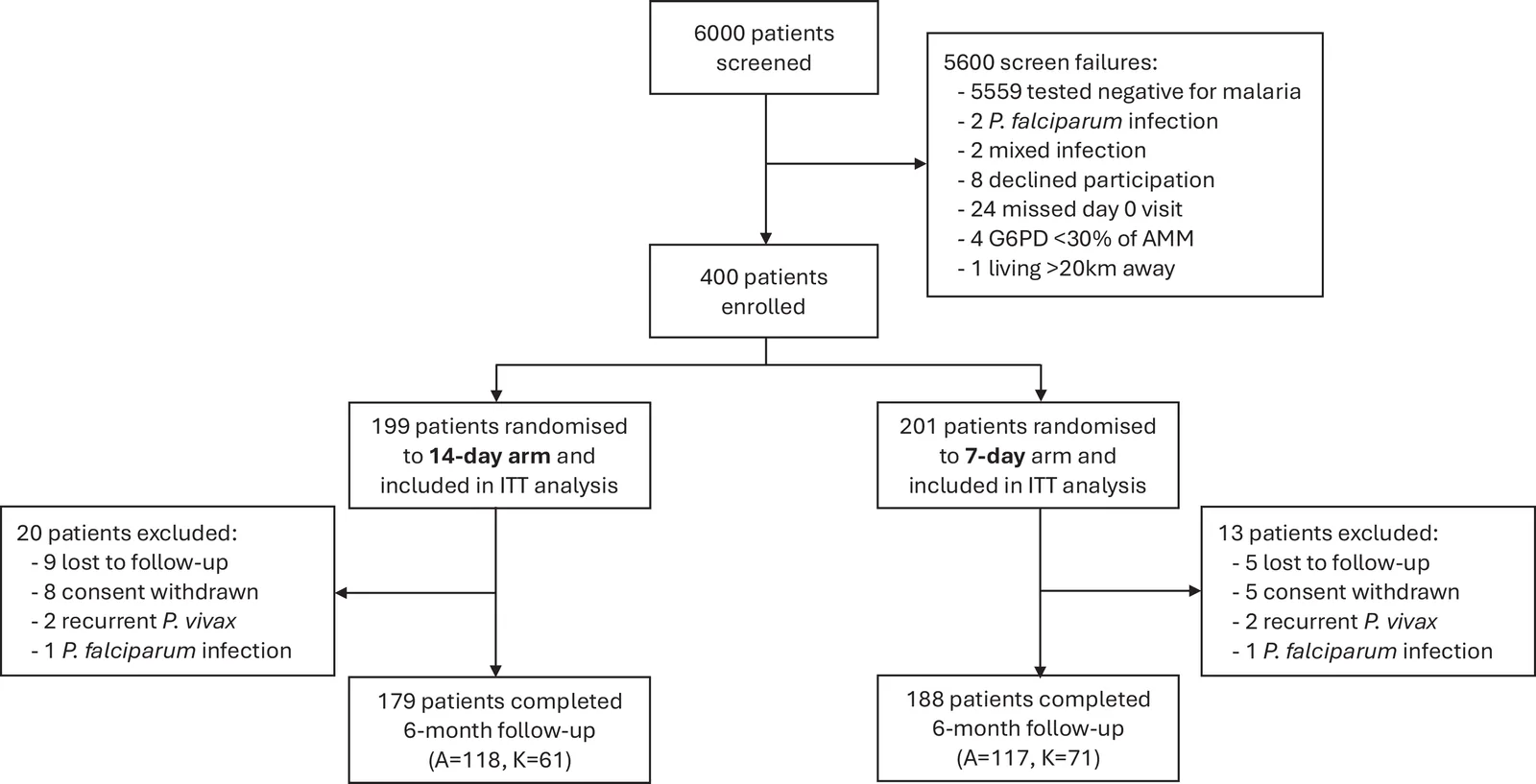
**Trial profile (consort)**. A, Ahmedabad; AMM, adjusted male median; ITT, intention to treat; K, Kolkata.

Baseline demographic and clinical characteristics were similar between the two groups, overall and within each study site (Table 1 and Supplementary File 3, pp 10-11). The median age of patients was 35 years (interquartile range [IQR] 25–46) and majority of patients were male (80·3% [321/400]). For patients in the 7-day group, the median daily dose of primaquine was 0·55 mg/kg (IQR 0·49–0·59; range 0·41–0·74) and for those in the 14-day group it was 0·27 mg/kg (IQR 0·24–0·29; range 0·20–0·37) (Supplementary File 3, p 14). Adherence to primaquine differed between the arms with 8% (16/201) recorded as having a missed dose in the 7-day group compared with 20% (40/199) in the 14-day group.

**Table 1:** Baseline characteristics of the intention-to-treat population.

	7 days PQ	14 days PQ
	N = 201	N = 199
Study site		
Ahmedabad	118/201 (59%)	118/199 (59%)
Kolkata	83/201 (41%)	81/199 (41%)
Age (years)	36·0 (25·0–47·0)	35·0 (25·0–45·0)
Self-reported sex		
Male	158/201 (79%)	163/199 (82%)
Female	43/201 (21%)	36/199 (18%)
Weight (kg)	59·0 (53·0–65·0)	58·5 (52·5–66·9)
Parasite density day −1 (/μL)	1831·6 (787·4–4916·9)	2387·4 (836·2–5580·0)
Body temperature day 0 (°C)	38·2 (36·2–39·1)	38·2 (36·2–38·8)
Presence of fever in the previous 48 h	199/201 (99%)	197/199 (99%)
Haemoglobin day 0 (g/dL)	12·3 (1·7)	12·1 (1·9)
G6PD activity (U/gHb)	13·0 (11·5–15·0)	13·0 (11·3–14·9)
Primaquine mg/kg daily dose	0·5 (0·5–0·6)	0·3 (0·2–0·3)

Data are presented as mean (SD) or median (IQR) for continuous measures, and n/total (%) for categorical measures.

By day 180, there were a total of four *P. vivax* recurrences, with two in each arm. In the 7-day group the recurrences occurred on day 58 and day 75, compared with day 127 and day 180 in the 14-day group. All four recurrences were symptomatic, and patients had received all expected doses of primaquine. The cumulative incidence of symptomatic parasitaemia by day 180 was 1·1% (95% CI 0·0–4·1) for the 7-day regimen and 1·1% (95% CI 0·0–4·4) for the 14-day regimen (hazard ratio 0·96, 95% CI 0·13–6·80, p = 0·97; Fig. 2). The cumulative incidence of symptomatic parasitaemia by day 180 for the 7-day regimen was within the predefined 10% non-inferiority margin compared with the 14-day regimen; results were identical for any parasitaemia by day 180 (Supplementary File 3, p 15). All four recurrences occurred at the site in Kolkata with a cumulative incidence of symptomatic parasitaemia by day 180 of 2·7% (95% CI 0·7–10·4) for the 7-day regimen and 3·2% (95% CI 0·8–12·1) for the 14-day regimen (hazard ratio 0·88, 95% CI 0·12–6·21, p = 0·89; Supplementary File 3, p 16). Results were similar for the per-protocol analysis, which excluded patients who missed a dose of primaquine and for analysis by sex (Supplementary File 3, pp 17–18).

**Fig. 2:**
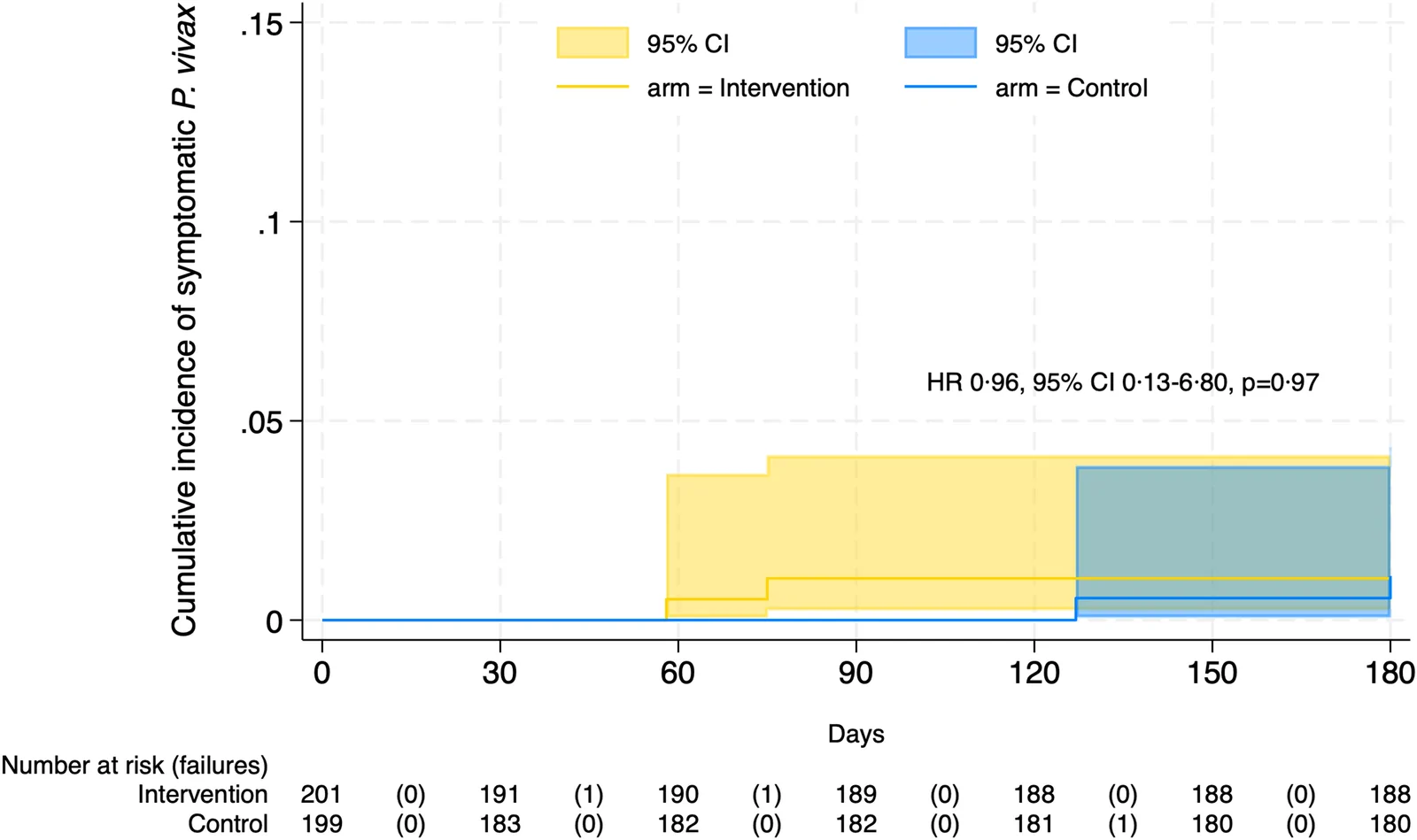
**Cumulative incidence of symptomatic parasitaemia by day 180.** Cumulative incidence of the time to first symptomatic parasitaemia between day 0 and day 180 in the intervention (7 days 0·5 mg/kg/day primaquine) and control (14 days 0·25 mg/kg/day primaquine) treatment arms. The hazard ratio (HR) and confidence interval (CI) compares the intervention to the control arm.

During 6 months of follow up 52 adverse events were reported in the 7-day group and 47 adverse events in the 14-day group. In both groups, most adverse events occurred within 28 days (67·3% [35/52] in the 7-day group and 74·5% [35/47] in the 14-day group). Overall, 11 adverse events in the 7-day group were possibly or probably related to the study drug compared with 13 events in the 14-day arm. All drug-related adverse events were mild, except for one moderate gastrointestinal event in the 14-day group.

There were two serious adverse events, both occurred in the 7-day group and were unrelated to the study drug. A 43-year-old female patient with a history of convulsions had a febrile convulsion 9 h after enrolment and was admitted to hospital for evaluation and treatment with anticonvulsants. The other serious adverse event was in a 17-year-old male who presented at the 4-month follow-up with pneumonia requiring admission to hospital and antibiotic therapy. Both patients made full recoveries.

None of the patients vomited primaquine within an hour of administration. On day 7 the risk of the gastrointestinal intolerance was 0·5% [1/183] in the 7-day group and 1·1% [2/180]) in the 14-day group. The corresponding risks on day 14 were also low (1·1% [2/179] and 0·6% [1/176] respectively; Table 2).

**Table 2:** Gastrointestinal symptoms (composite^a^) at day 0, 2, 7 and 14 by treatment arm.

Day	7-day primaquine	14-day primaquine
0	94/201 (46·8%)	79/199 (39·7%)
2	43/192 (22·4%)	31/186 (16·7%)
7	1/183 (0·5%)	2/180 (1·1%)
14	2/179 (1·1%)	1/176 (0·6%)

a The composite gastrointestinal outcome included the presence of any of abdominal pain, diarrhoea, nausea, vomiting or anorexia.

The risk of clinically significant haemolysis was low in both groups. No patients required blood transfusions or had a haemoglobin fall from baseline by >5 g/dl within 14 days. One patient in the 14-day group had a fall in haemoglobin to <8 g/dL (from 8·9 g/dL at baseline to 6·7 g/dL on day 1), but by day 7, it had recovered to 8·7 g/dL. One patient in each treatment arm had a >25% fall between day 0 and days 1–14 (from 13·5 g/dL to 9·2 g/dL and from 13·4 g/dL to 10·0 g/dL; Table 3).

**Table 3:** Serious haemolytic outcomes by treatment arm.

Outcome	7-day primaquine	14-day primaquine
Develop anaemia by days 1–14 (Hb < 8 g/dL)	0/194 (0·0%)	1/194 (0·5%)
Blood transfusion	0/201 (0·0%)	0/199 (0·0%)
Hb decreased by >25% by days 1–14	1/194 (0·5%)	1/194 (0·5%)
Hb drop by >5 g/dL by days 1–14	0/194 (0·0%)	0/194 (0·0%)

Hb, haemoglobin.

## Discussion

Our study demonstrates that a 3·5 mg/kg total dose primaquine regimen administered over 7 days for the radical cure of *P*. *vivax* malaria in India is non-inferior within 180 days post treatment initiation compared to a regimen administered over 14 days. It is also safe and well tolerated in patients with normal (≥30%) G6PD activity. The risk of recurrence was low in both treatment groups, however, substantially fewer recurrences than anticipated limited the precision of the efficacy estimates. Studies with follow up longer than 6 months could provide additional data to establish efficacy of the regimen against late relapses. Adverse events were few and mild, and no clinically significant safety concerns were detected. These findings provide important evidence for optimising radical cure strategies in India, where *P. vivax* remains a substantial contributor to malaria burden and effective treatment options are essential for progress towards malaria elimination.

Adherence to prolonged primaquine treatment regimens remains a major challenge, and non-adherence under unsupervised conditions leads to poor effectiveness.^6^^,^^7^ Shorter treatment regimens are likely to improve adherence and therefore effectiveness.^16^ Based on this, the 2022 updates of the WHO Malaria Treatment Guidelines recommended administering the same total low dose at 0·5 mg/kg for 7 days rather than 0·25 mg/kg for 14 days, assuming that patients were screened for G6PD deficiency.^9^ Our findings support this global recommendation. The 7-day low-dose regimen had comparable efficacy and safety to the 14-day regimen, underscoring its potential to improve adherence and operational feasibility in India. In addition, adherence was greater in patients treated with the 7-day regimen, of whom 8% missed a primaquine dose compared with 20% in those treated with the 14-day regimen. However, this difference may reflect patients in the 7-day arm receiving more days of supervised primaquine (3 days or 43% of all doses) compared with the 14-day arm (4 days or 29% of all doses).

Accumulating global evidence highlights that higher total doses of primaquine improve anti-relapse efficacy.^11^ Recent individual patient data meta-analyses informed the 2024 WHO malaria guideline revision, which now recommends a higher total primaquine dose of 7 mg/kg administered over 7 or 14 days to ensure adequate radical cure.^10^ However, the benefit of higher doses is less pronounced in South Asia, where the overall risk of relapse is significantly lower than in South America and South East Asia.^2^ Our data confirms that a low-dose primaquine regimen over 7 or 14 days is associated with a low risk of relapse within 6 months in this region.^5^

Shorter primaquine regimens require a higher daily dose to achieve the same total dose, and this increases the risk of haemolysis in individuals with reduced G6PD enzyme activity. Reliable G6PD screening is thus a prerequisite for safely implementing condensed dosing schedules with daily doses higher than 0·5 mg/kg. In our study, eligibility for primaquine was determined using a quantitative test with a 30% activity threshold, consistent with WHO recommendations.^10^ India has a heterogeneous and large burden of G6PD deficiency, although the overall prevalence is low (1·9%) there is substantial geographic variation between populations, varying from 0·8% to 6·3%,^21^ with higher frequencies reported among tribal populations.^22^ Multiple G6PD variants circulate within Indian populations, each associated with different levels of residual enzyme activity and, associated risks of drug-induced haemolysis.^21^ The most commonly reported G6PD variants are the Mediterranean and Orissa variants, which together account for approximately 80% of G6PD deficiency cases.^21^

At the two study sites of this study, the prevalence of G6PD deficiency is estimated to be 1·8% in Ahmedabad^23^ and 1·4% in urban Kolkata.^24^ In our study, none of the patients had clinically significant haemolysis or severe reductions in haemoglobin, suggesting that low-dose regimens administered with appropriate G6PD screening can be safely delivered over 7 or 14 days.

The current WHO antimalarial guidelines recommend assessing patients’ G6PD activity with a qualitative test prior to prescribing daily primaquine doses of 0·25–0·5 mg/kg.^10^ Qualitative assays assess patients at the 30% activity threshold, and identify patients with severe G6PD deficiency who should not be prescribed a daily primaquine regimen. Since none of the available qualitative tests are WHO pre-qualified, we measured activity in our study using the SD Biosensor STANDARD G6PD™ test (SD Biosensor), a semi-quantitative assay, and by spectrophotometry, the gold standard quantitative assay. The STANDARD G6PD test was developed to support the implementation of tafenoquine and to identify individuals with intermediate enzyme activity (30–70% activity).^25^

India is currently exploring whether to introduce tafenoquine into national guidelines; this single-dose regimen for the radical cure of *P. vivax* malaria overcomes poor adherence to daily primaquine regimens and thus has the potential to improve anti-relapse effectiveness. A trial of tafenoquine versus primaquine is currently underway in India (NCT06666491). The evidence from our trial provides a useful intermediary model. Integrating routine quantitative G6PD testing with a simplified primaquine regimen offers a pragmatic approach for programme readiness. During a transition period, before tafenoquine is potentially introduced, adopting a 7-day primaquine schedule supported by point-of-care G6PD testing could accelerate national progress toward elimination targets.

Both the 7- and 14-day low-dose primaquine regimens were well tolerated. Adverse events were predominantly mild and were similar between treatment arms. Gastrointestinal symptoms, a common tolerability concern with primaquine, were comparable between treatment arms. Gastrointestinal discomfort has been shown to increase with higher daily doses of primaquine, although this can often be mitigated by co-administration with food.^26^^,^^27^ Previous pooled analyses have demonstrated that the 0·5 mg/kg daily dose is not associated with an increased risk of gastrointestinal symptoms in adults, with significant increases only observed at the 1 mg/kg daily dose.^11^

This trial has several limitations. First, the trial was open-label, preventing a blinded assessment of the effect of different trial arms. Second, the sample size was modest and the overall number of recurrences was much lower than anticipated, limiting the detection of differences in efficacy and increasing the probability that a potential true difference between the treatment arms would not be identified. Third, although follow-up was rigorous, not all doses were supervised, implying that adherence was likely reduced compared to a fully supervised trial, but likely better compared to routine care. The higher proportion of patients with missed doses in the 14-day arm highlights the difficulties of giving a prolonged course of primaquine, even in a trial setting. Fourth, the study included only patients older than 16 years from two study sites. Findings cannot be extrapolated to children and may not be generalisable to all settings in India. Fifth, the 6 month follow-up period is consistent with prior trials, but may have underestimated the true risk of relapses, since a substantial proportion of *P. vivax* recurrences occur after 6 months in India.^2^ Trials from the Indian subcontinent have demonstrated a median time to recurrence of 120 days,^28^ but there has been substantial heterogeneity with some showing a high proportion of recurrences occurred before 6 months.^29^^,^^30^ The lack of a control arm without radical cure prevents a direct assessment of the efficacy of primaquine in either arm in this study, however, in a meta-analysis from South Asia the expected risk of recurrence without primaquine after 6 months was found to be 12·1% (95% CI 7·7–17·2),^5^ suggesting a moderate benefit of both the 7- and 14-day primaquine regimens when compared to historical data within 180 days. Sixth, the Kaplan–Meier survival analysis used in the study assumes that loss to follow up, and censoring of patients, was random and unrelated to the treatment. The overall degree of loss to follow up was low and thus subsequent censoring was similar between treatment arms. Therefore, whilst the risk of bias due to treatment is likely to be low, we cannot rule out unidentified biases. Finally, the primary outcome used was *P. vivax* recurrence rather than relapse, as relapses can be genetically related or unrelated to the original infection, which prevents recurrence aetiology from being determined definitively by genomic methods. However, this approach ensures that our primary endpoint was conservative and, if anything, it will have underestimated anti-relapse efficacy.^31^

In conclusion, this trial provides evidence from India on the 6 month efficacy, safety and tolerability of the low dose (3·5 mg/kg total dose) 7-day primaquine regimen for *P. vivax* radical cure. Our findings support the WHO guidance and could further inform India's national treatment guidelines for *P. vivax* infections. Given the potential for relapse beyond the 180-day follow-up period, studies with longer follow up could provide additional data to establish the efficacy of this regimen post 6 months.

## Contributors

RKB, SSP, SMN, MR, AA, PJG, RNP, MH, PKB, KT, and RJC conceived the study. PJG gained study funding. RKB, SSP, SMN, RC, SKP, PHR, BS, KJU, LG, HM, HK, SRC, AC, SG, SSP, DS, PKB, and NN were involved in data collection. SSP, RC, MSH, KY, RV, GL, HM, AA, PJG, MR, PKB, KT, NN, and RJC were involved in study oversight and training. SSP, AG, RC, MSH, MR, PKB, KT, NN, and RJC conducted data curation, analysis, and interpretation. AG, MSH, and RJC have accessed and verified the data. RKB, SSP, SMN, RC, PJG, PKB, KT, NN, and RJC developed the first draft of the manuscript. All authors reviewed and approved the final version of the manuscript. KT, NN, and RJC were responsible for the decision to submit the manuscript.

## Data sharing statement

Data from this analysis can be accessed by sending an email with appropriate justification to the corresponding author at the National Institute of Malaria Research. Data requests can also be made through the WWARN website (https://www.wwarn.org/). Data access requests will be reviewed by the Data Access Committee to ensure compliance with ethical approvals and principles of equitable data sharing. Requests can be submitted by email to malariaDAC@iddo.org via the data access form available at https://www.wwarn.org/working-together/sharing-accessing-data/accessing-data. WWARN is registered with the Registry of Research Data Repositories (https://www.re3data.org/).

## Declaration of interests

RJC, RNP, and KT were supported by the Australian National Health and Medical Research Council (NHMRC) Investigator Grants (1194702, 2008501, and 2033264 respectively). RJC is supported by the Royal Australasian College of Physicians (RACP) Cottrell Research Establishment Fellowship from the RACP Foundation and Dr Jack Dinham Cottrell. MR is supported by the Australian NHMRC CRE Grant 2024622 (Australian Centre of Research Excellence for Malaria Elimination) and Australian NHMRC Synergy Grant 2018654. All other authors have no conflicts of interest to declare.

## Appendix A Supplementary data

Study Protocol

Statistical Analysis
