# Supplementary material for: Seven-day versus fourteen-day low-dose primaquine for Plasmodium vivax malaria in India: an open-label, non-inferiority, randomised, controlled trial
Source: Lancet Reg Health Southeast Asia. 2026 Aug 25;53:100849. doi: 10.1016/j.lansea.2026.100849 (PMC13651684; doi:10.1016/j.lansea.2026.100849)
Supplement: Supplementary file 1 [file mmc1.pdf]

Trials

STUDY PROTOCOL

Open Access

A randomised controlled trial to compare the efficacy, safety, and tolerability of low dose, short course primaquine in adults with uncomplicated *P. vivax* malaria in two hospitals in India

Sundus Shafat Ahmad[1†], Reena Verma[1†], Robert J. Commons[2,3,4], Nitika[1], Sauman Singh‑Phulgenda[5,6], Rutuja Chhajed[5,6], Praveen K. Bharti[1], Beauty Behera[7], Syed Mohammad Naser[8], Salil Kumar Pal[8], Parinita Halder Ranjit[8], Rajendra Kumar Baharia[9], Bhavin Solanki[10], K. J. Upadhyay[11], Philippe J. Guerin[5,6], Amit Sharma[12], Ric N. Price[2,3,6,13], Manju Rahi[14,15*] and Kamala Thriemer[3*]

Abstract

Background *Plasmodium vivax* remains a major challenge for malaria control and elimination due to its ability to cause relapsing illness. To prevent relapses the Indian National Center for Vector Borne Diseases Control (NCVBDC) recommends treatment with primaquine at a dose of 0.25 mg/kg/day provided over 14 days. Shorter treatment courses may improve adherence and treatment effectiveness.

Methods This is a hospital-based, randomised, controlled, open-label trial in two centres in India. Patients above the age of 16 years, with uncomplicated vivax malaria, G6PD activity of ≥ 30% of the adjusted male median (AMM) and haemoglobin levels ≥ 8 g/dL will be recruited into the study and randomised in a 1:1 ratio to receive standard schizonticidal treatment plus 7-day primaquine at 0.50 mg/kg/day or standard care with schizonticidal treatment plus 14-day primaquine at 0.25 mg/kg/day. Patients will be followed up for 6 months. The primary endpoint is the incidence risk of any *P. vivax* parasitaemia at 6 months. Safety outcomes include the incidence risk of severe anaemia (haemoglobin < 8 g/dL), the risk of blood transfusion, a > 25% fall in haemoglobin and an acute drop in haemoglobin of > 5 g/dL during primaquine treatment.

Discussion This study will evaluate the efficacy and safety of a 7-day primaquine regimen compared to the standard 14-day regimen in India. Results from this trial are likely to directly inform national treatment guidelines.

Trial registration Trial is registered on CTRI portal, Registration No: CTRI/2022/12/048283.

Keywords *P. vivax*, Vivax malaria, Radical cure, Primaquine, Malaria elimination, Randomised controlled trial

†Sundus Shafat Ahmad and Reena Verma are joint first authors.

*Correspondence:
Manju Rahi
drmanjurahi@gmail.com
Kamala Thriemer
kamala.ley-thriemer@menzies.edu.au
Full list of author information is available at the end of the article

© The Author(s) 2024. **Open Access** This article is licensed under a Creative Commons Attribution 4.0 International License, which permits use, sharing, adaptation, distribution and reproduction in any medium or format, as long as you give appropriate credit to the original author(s) and the source, provide a link to the Creative Commons licence, and indicate if changes were made. The images or other third party material in this article are included in the article's Creative Commons licence, unless indicated otherwise in a credit line to the material. If material is not included in the article's Creative Commons licence and your intended use is not permitted by statutory regulation or exceeds the permitted use, you will need to obtain permission directly from the copyright holder. To view a copy of this licence, visit http://creativecommons.org/licenses/by/4.0/. The Creative Commons Public Domain Dedication waiver (http://creativecommons.org/publicdomain/zero/1.0/) applies to the data made available in this article, unless otherwise stated in a credit line to the data.

Administrative information

Note: the numbers in curly brackets in this protocol refer to SPIRIT checklist item numbers.

Title {1}	A randomised controlled trial to compare the efficacy, safety, and tolerability of low dose, short course primaquine in adults with uncomplicated *P. vivax* malaria in two hospitals in India
Trial registration {2a and 2b}	CTRI Registration No. CTRI/2022/12/048283<br>Item 2b: Not applicable
Protocol version {3}	Version 3.0, March 2023
Funding {4}	Infectious Diseases Data Observatory (IDDO), University of Oxford, New Richards Building, Old Road Campus, Roosevelt Drive, Oxford, OX3 7LG, UK
Author details {5a}	1. ICMR-National Institute of Malaria Research, New Delhi, India<br>2. WorldWide Antimalarial Resistance Network, Asia-Pacific Regional Hub, Australia<br>3. Menzies School of Health Research and Charles Darwin University, Darwin, Australia<br>4. General and Subspecialty Medicine, Grampians Health, Ballarat, Australia<br>5. Infectious Diseases Data Observatory – IDDO, Oxford, OX3 7LG, UK<br>6. Centre for Tropical Medicine and Global Health, Nuffield Department of Medicine, University of Oxford, New Richards Building, Old Road Campus, Roosevelt Drive, Oxford, OX3 7LG, UK<br>7. Delhi Skill and Entrepreneurship University, New Delhi, India<br>8. Calcutta National Medical College, Kolkata, West Bengal, India<br>9. NIMR Field Unit, Kheda, Gujarat, India<br>10. Ahmedabad Municipal Corporation, Ahmedabad, Gujarat, India<br>11. BJ Medical College, Ahmedabad, Gujarat, India<br>12. International Centre for Genetic Engineering and Biotechnology, New Delhi, India<br>13. Academy of Scientific and Innovative Research (AcSIR), Ghaziabad, India<br>14. Mahidol-Oxford Tropical Medicine Research Unit, Faculty of Tropical Medicine, Mahidol University, Bangkok, Thailand.<br>15. Indian Council of Medical Research, New Delhi, India
Name and contact information for the trial sponsor {5b}	ICMR-National Institute of Malaria Research, New Delhi, India
Role of sponsor {5c}	The study sponsor has full authority on study design, data collection, management, analysis, and interpretation of data, writing of the report, and the decision to submit the report for publication.

Introduction

Background and rationale {6a}

In 2021, there were an estimated 247 million cases of malaria from 84 malaria-endemic countries, out of which 4.9 million were caused by *Plasmodium vivax* [1]. India accounted for 79% of cases in the South East Asia (SEA) region and reported approximately 60,000 vivax malaria cases [1]. India along with Pakistan, Papua New Guinea and Ethiopia are the largest contributors to the global vivax burden [1]. There has been a considerable decrease in the malaria burden over the last 6 years since India launched its plan for malaria elimination in 2016; however, case numbers for of vivax malaria remain high. In 2022, the number of cases of vivax malaria increased to 74,126, accounting for 43% of all cases of malaria [2]. If India is to reach its target for malaria elimination in 2030, better strategies will be needed to address the *P. vivax* burden [3].

P. vivax is a substantial challenge for malaria control and elimination because of its ability to cause relapsing infections. Relapse results from the activation of dormant liver-stage hypnozoites and can occur weeks or months following an acute infection of vivax malaria [4]. Recurrent infections are associated with a febrile illness, an increased cumulative risk of severe anaemia, direct and indirect mortality, and are an important source of onward transmission of the parasite [4]. The only widely available hypnozoitocidal drug (capable of clearing the intra-hepatic hypnozoites of *P. vivax*) is primaquine. Primaquine is an 8-aminoquinoline compound with the potential of causing severe drug-induced haemolysis in glucose-6-phosphate-dehydrogenase (G6PD) deficient patients. Novel point-of-care tests for G6PD deficiency are now available [5] and they have the potential to facilitate the safer use of primaquine.

The Indian National Center for Vector Borne Diseases Control (NCVBDC) recommends primaquine for the radical cure of vivax malaria in the country. The guidelines recommend caution in areas with a known high prevalence of G6PD deficiency and patients are advised to stop the drug intake if adverse effects occur [6, 7]. To reduce the risk of drug-induced adverse effects, primaquine is recommended at a total dose of 3.5 mg/kg given over 14 days (0.25 mg/kg per day) [7]. However, this prolonged treatment is associated with poor compliance and therefore low effectiveness [8]. To ensure better adherence to the 14-day primaquine regimen, supervised administration of primaquine can be effective and considered to enhance compliance among patients with vivax malaria in India [9]. However,

directly observed treatment will require additional costs. An alternative option to increase adherence is a shorter treatment regimen such as a 7-day primaquine course (0.5 mg/kg day over 7 days) which was recently endorsed by WHO [10].

There is limited evidence for the efficacy and safety of this shorter regimen from India and South Asia in general. Two studies have been conducted, one including a sustained release formulation of primaquine [11], and the other was conducted more than 10 years ago [12]. An individual patient data meta-analysis has demonstrated a lack of available evidence on optimal primaquine dosing in South Asia supporting the need for additional data on 7-day primaquine efficacy and safety in India [13].

Our open-label randomised trial aims to compare the efficacy, tolerability and safety of short-course, low-dose (0.50 mg/kg/day for 7 days) primaquine, with the current standard of care (0.25 mg/kg/day for14 days) to prevent *P. vivax* recurrences in India.

Objectives {7}

- To assess the 6-month efficacy of a 7-day course of primaquine treatment regimen (0.50 mg/kg/day) in preventing recurrent *P. vivax* parasitaemia in G6PD normal patients over 16 years of age compared to the current 14-day primaquine regimen (0.25 mg/kg/day)
- To assess the safety and tolerability of a 7-day course of primaquine treatment regimen compared to the current standard primaquine regimen.

Trial design {8}

This is a randomised, controlled, non-inferiority, open-label trial in G6PD normal patients with uncomplicated *P. vivax* malaria comparing a shorter duration primaquine regimen to the current standard primaquine treatment to prevent vivax recurrence.

Methods: participants, interventions and outcomes

Study settings {9}

Two sites have been selected in India. The first site is Calcutta National Medical College, Kolkata, West Bengal and the second site is Ahmedabad Municipal Corporation Hospital, Ahmedabad, Gujarat.

Eligibility criteria {10}

Inclusion criteria

The participant may enter the study if all of the following apply:

- *P. vivax* (asexual) malaria mono-infection confirmed by microscopy on a thick or thin blood smear.
- Age 16 years and over of either gender
- Fever > 37.5 °C axillary, or a history of fever within the previous 48 h.
- Female patients of child-bearing potential will be included if non-lactating and willing to use contraceptive methods during the study period. Non-child-bearing potential is defined as:

  Post-menopausal (12 months of spontaneous amenorrhea or 6 months of spontaneous amenorrhea with serum FSH > 40 mIU/mL), pre-menopausal and has had a hysterectomy or a bilateral oophorectomy or a bilateral tubal ligation with medical report verification or,

  Child-bearing potential, has a negative urine pregnancy test at screening, and agrees to comply with one of the following during the treatment stage of the study and for a period of 90 days after stopping the study drug:

  a. Use of oral contraceptive, either combined or progesterone alone (if no contraindications to oral contraceptives exist).
  b. Barrier contraceptive if oral contraceptives are contraindicated.
  c. Use of an intrauterine device.

- Willing to give informed consent.
- Willing to comply with protocol instructions and duration of follow-up.
- Living in and around the site facilities (to facilitate follow-up).

Exclusion criteria

The participant may not enter the study if any of the following apply:

- Patients with G6PD activity less than 30% of the adjusted male median (AMM), tested by UV spectrophotometry.
- Patients with mixed infection with *P. vivax* and *P. falciparum* (e.g. identified by Giemsa-stained smear or rapid diagnostic test).
- Patients with dengue coinfection (detected by dengue NS1 antigen rapid test).
- Patient with body weight less than 40 kg.
- Severe *P. vivax* malaria is defined as per National Guidelines [6] and WHO[9] criteria.
- Haemoglobin <8 g/dL.

- History of haemolytic episodes or haemolytic anaemia or methaemoglobinemia or blood transfusion within the past 90 days.
- Unable to tolerate oral treatment.
- Pregnant and lactating women.
- Known allergy to chloroquine, primaquine or any other related drugs.
- Evidence of gastro-intestinal dysfunction that could alter absorption or motility (e.g. diarrhoea defined as >3 episodes of watery stools in the previous 24 h or patients who have had three episodes of vomiting within 24 h prior to screening).
- Use of concomitant medications that could induce haemolysis or haemolytic anaemia or depressants of myeloid element of the bone marrow.
- Any antimalarial treatment taken during 1 month prior to screening.
- Ongoing prophylaxis with drugs having antimalarial activity.
- Participation in any other investigational drug study of within 3 months prior to screening.
- Any other underlying disease that would require hospitalisation and could compromise the diagnosis and the evaluation of the response to the study medication (including clinical symptoms of immunosuppression, HIV, hepatitis, tuberculosis, splenectomy conducted earlier as confirmed by history or clinical examination; evidence of clinically significant cardiovascular, pulmonary, metabolic, gastrointestinal, neurological, or endocrine diseases, malignancy, or other abnormalities).
- Retinal/visual field defects or auditory defects and history of psoriasis and porphyria. Subjects meeting any of the exclusion criteria will not be enrolled in the study and treated according to NCVBDC Guidelines.

Who will take informed consent? {26a}

Patients attending the hospitals at either study site will be screened for enrolment into the trial. On screening day (Day − 1), the patients will be provided with a brief written consent outlining the screening tests to be conducted and once a patient qualifies for enrolment, a complete informed consent process will be initiated before enrolment (Day 0). The informed consent describing the purpose of the study, the procedures to be followed, and the risks and benefits of participation will be sought from the participants prior to enrolment. The age of consent will be considered as ≥ 18 years. For participants between the age of 16–18, written assent will be obtained. The signed written informed consent will be obtained by trained and authorised staff from each subject prior to enrolment in the study. For adolescents who are not legally able to give consent, written informed consent is obtained from their Legally Authorised Representative (LAR) in accordance with applicable laws or regulations. All interviews will be conducted in the native language (Gujarati, Bengali or Hindi) of the patients by the study staff. If a patient, parent or guardian is unable to read or write, a signature from a witness to the informed consent discussion will be obtained. Patients/parents or guardians will be informed that participation in the study is completely voluntary and that they may withdraw from the study at any time.

Additional consent provisions for collection and use of participant data and biological specimens {26b}

All patients will be asked to provide consent for the long-term storage of blood samples in form of dried blood spots (DBS) for molecular analysis of the parasite. Furthermore, patients are informed about the use of their health data for current and any further research in relation to it at enrolment.

Interventions

This trial consists of two arms:

- Intervention arm: 0.50 mg/kg/day primaquine for 7 days (3.5 mg/kg total dose)
- Control arm: 0.25 mg/kg/day primaquine for 14 days (3.5 mg/kg total dose)

Additionally, all participants in both arms will receive blood schizonticide treatment, i.e. chloroquine (total dose 25 mg base/kg) as per NCVBDC guidelines [6, 7]. All chloroquine doses will be directly supervised.

All patients will be tested initially, and those found to be G6PD deficient (defined as < 30% of AMM) will be excluded from this randomised study but will receive medical care according to WHO guidelines: 0.75 mg/kg/week for 8 doses (total dose 6 mg/kg).

Explanation for the choice of comparators {6b}

All participants in the control arm will be treated with radical cure for acute uncomplicated vivax malaria according to local NCVBDC guidelines [6, 7]: 0.25 mg/kg bodyweight primaquine for 14 days, starting on enrolment (Day 0). If the participant vomits the treatment within 60 min, a repeat dose will be administered. If the patient vomits the second dose, he or she will receive parenteral therapy with artesunate and will be withdrawn from the study. Primaquine treatment will be observed daily on the first 3 days, and then alternatively up to 14 days by home visits by staff. Primaquine will be administered with food, to help reduce gastrointestinal side effects.

Intervention description {11a}

Patients in the intervention arm will be treated with a 3 day regimen of chloroquine (total dose 25 mg base/kg) plus primaquine (0.50 mg/kg bodyweight for 7 days). If the participant vomits the treatment within 60 min, a repeat dose will be administered. If the patient vomits the second dose, he or she will receive parenteral therapy with an artemisinin combination therapy (ACT) and be withdrawn from the study. Primaquine treatment will be observed daily on the first 3 days, and then on alternate days up to 7 days by home visits by staff. Primaquine will be administered with food, to help reduce gastrointestinal side effects.

Criteria for discontinuing or modifying allocated interventions {11b}

Each participant has the right to withdraw from the study at any time. If the participant withdraws consent, uses antimalarial drugs outside of the study protocol, or who fails to attend a follow-up visit and is unable to be located within 48 h on days 1–14 or for more than 2 subsequent visits post day 14, the patient will be excluded/withdrawn. It will be left to the investigator's clinical judgment whether or not an adverse event (AE) is of sufficient severity to require stopping the participant's treatment. If the participant is withdrawn due to an AE, the investigator will arrange for follow-up visits or telephone calls until the AE has resolved and conditions stabilised.

Rescue treatment

Patients who do not respond rapidly to treatment will be administered rescue medication. If a patient vomits twice, he or she will receive parenteral therapy with artesunate and will be withdrawn from the study. Patients with signs of severe or complicated malaria will be hospitalised and treated with parenteral therapy and relevant supportive treatment. If a patient meets one of the criteria for therapeutic failure, he or she will be treated according to current national recommendations.

Strategies to improve adherence to interventions {11c}

Patients of both arms will be visited at home by study staff on alternate days and provided with medication for 2 days. On the day of visit, primaquine doses will be observed and the patient is advised to take treatment on the following day. The patient will also be asked about the previous day's dose. If patients are not found/located, all efforts will be made to ensure that they restart treatment.

Relevant concomitant care permitted or prohibited during the trial {11d}

At enrolment, all anti-malarial medications received in the preceding 28 days will be documented on the clinical record form (CRF). Regular medication at trial entry for conditions other than malaria will be documented. Patients will be asked to continue to take any regular medications in their usual prescriptive way. Additional drugs taken during the trial period for whatever reason will be documented. Patients discontinuing their trial medication prematurely, or who fail to respond to trial medication and receive other anti-malarial therapy, will be recorded with a start and end date. Intake of drugs with antimalarial activity will be discouraged, unless prescribed by the medical officer of the trial.

Provisions for post-trial care {30}

The sponsor has a provision of insurance, which is in accordance with the legal requirements. This insurance provides coverage for damage to research subjects through injury or death caused by any activities of the study.

Outcomes {12}

Primary outcomes

Efficacy

- The incidence risk of symptomatic *P. vivax* malaria at 6 months.
- A participant will be considered to have demonstrated recurrence-free efficacy at 6 months if:

  The participant had *P. vivax* asexual parasitaemia at enrolment.

  The participant has initial clearance of *P. vivax* parasitaemia defined as two negative blood films for asexual P. vivax parasites, with at least 6 h between the counts, and no positive blood films in the interval.

  The participant was aparasitaemic for asexual *P. vivax* at any assessment following initial parasite clearance.

  The participant did not take a concomitant medication with anti-malarial activity at any point between the initial study day and their last parasite assessment.

  The participant is parasite-free at the end of 6 months.

Secondary efficacy outcome

The incidence risk of any (symptomatic and asymptomatic) *P. vivax* malaria at day 28 and at 6 months.

Safety

- The proportion of patients vomiting their medication within 1 hour of administration.
- The proportion of adverse events and serious adverse events.
- The incidence risk of anaemia (Hb < 8 g/dL) and/or the risk for blood transfusion.
- Risk of > 25% fall in haemoglobin on any day of treatment.
- The incidence risk of an acute drop in Hb of > 5 g/dL during primaquine treatment.
- Gastrointestinal (GI) tolerability — incidence of abdominal pain, heartburn, diarrhoea, constipation, nausea and vomiting.

Participant timeline {13}

The participant timeline can be found in Table 1.

Sample size {14}

The 0.25mg/kg/day × 14 days regimen of primaquine is associated with ~13% recurrence rate of vivax malaria [12]. The present study will assess if 0.50 mg/kg/day for 7 days will have an equivalent recurrence rate within a margin of 10%. A sample size of 200 per group, assuming a 30% loss to follow-up at 5% significance will provide 68% power to detect this difference. Accordingly, we have decided on 200 patients at each site (100 in each arm), 400 in total.

Recruitment {15}

Trial participants

Male and female patients over the age of 16 years, with uncomplicated vivax malaria presenting to a participating centre will be enrolled into the study if they fulfil the eligibility criteria. Patients will be recruited at established hospital sites in malaria-endemic areas with large catchment areas and sufficient malaria patient load. Patients will be recruited for a period of 6 months and it is aimed to recruit an equal number of participants at both sites.

Methods: assignment of interventions

Sequence generation {16a}

Patients presenting with *P. vivax* malaria, who are G6PD normal and meet the other eligibility criteria will be allocated randomly to one of two arms. The allocation ratio of is 1:1, and randomisation will be performed using computer-generated random numbers. The randomisation sequence will be generated by an independent statistician who is not involved in the enrolment or assignment of participants. Information on block size has not been shared with the team recruiting patients.

Concealment mechanism {16b}

To ensure allocation concealment, the randomisation list will be securely stored in the study database by an independent team member with no direct involvement in patient recruitment or assessment. The next allocation will remain concealed until the screening process is complete, and the patient qualifies for enrolment in the study.

Implementation {16c}

Enrolment and assignment of participants will be done by qualified study staff who have received delegation by the study PI to conduct these tasks. The allocation sequence, generated by the independent statistician, will be kept confidential and will not be accessible to the study staff involved in participant enrolment and assignment. The study staff will only have access to the allocation information at the time of participant assignment.

Assignment of interventions: blinding

Who will be blinded {17a}

Not applicable, as neither participants nor study staff will be blinded to the allocation.

Procedure for unblinding if needed {17b}

Not applicable, as the study is not blinded, therefore no procedures for unblinding are required.

Data collection and management

Plans for assessment and collection of outcome {18a}

Data will be collected as described below and in the study Table 1. All study data will be recorded on standard Case Report Forms (CRF) and entered into a REDCap database, a GCP-compliant data management system.

Pre-study

Only patients with G6PD activity ≥30% of the adjusted male median (AMM) as determined by UV spectrophotometry will be eligible for enrolment into the main trial. In order to determine 100% G6PD activity at each site, a total of 30 adult male healthy volunteers from the health facility will be sampled. A separate written informed consent will be requested. A venous blood sample (3–5 ml) will be collected and G6PD activity measured using UV Spectrophotometry. No follow-up of those participants will be required.

Table 1 Study table

	Screening (Day − 1)	Enrolment (Day 0)	Day 1	Day 2	Day 3–6	Day 7	Day 8–13	Day 14	Day 21	Day 28	Month 2	Month 3	Month 4	Month 5	Month 6	Day of recurrence
Treatment																
CQ	X	X	X													
14 day PQ 0.25 mg/kg BW		X	X	X	X	X	X									
7 day arm PQ 0.50 mg/kg BW		X	X	X	X											
Procedures																
Malaria RDT for *P. vivax*	X															X
Dengue NS1 RDT	X															X
Physical examination including temp		X	X	X		X		X	X	X	X	X	X	X	X	X
CBC and LFT		X														X
G6PD UVS	X															
G6PD Biosensor	X															
Microscopy	X	X	X	X		X		X	X	X	X	X	X	X	X	X
Hb by Hemocue		X	X	X		X		X	X	X	X	X	X	X	X	X
Urine pregnancy test	X															X
Physical examination of urine			X	X		X										X
DBS		X														X
AE/SAE		X	X	X	X	X	X	X	X	X	X	X	X	X	X	X

Study procedures for the main study

The screening process will be divided over the period of 2 days (Day − 1 and Day 0), and so will be the process of informed consent. This will be done to accommodate for potential delays in receiving the screening results for the G6PD testing. On Day − 1, a brief informed consent will be taken for initial screening tests and if the participant qualified for enrolment, written informed consent will be taken for enrolment into trial purposes. Detailed procedures are listed in Table 1.

Day − 1: All patients coming to the hospital with fever will be screened. For screening purposes, a brief informed consent will be taken and a venous blood sample will be collected. This will be used for malaria RDT, blood film microscopy, quantification of parasite density, dengue NS1 screening and G6PD testing. Women between the ages of 16 to 49 years willing to participate will be asked to take a urinary pregnancy test. A clinical checklist will be used to evaluate the exclusion criteria. Patients who test positive for vivax malaria will receive their first dose of chloroquine.

Day 0: Participants diagnosed with G6PD deficiency will be informed of their status and will be provided with information on the diagnosis and its consequences in everyday life and referred to a regular hospital clinician for treatment. Haemoglobin will be screened using Hemocue™ machine and patients who meet all enrolment criteria and are willing to provide written informed consent will be enrolled into the trial. Following enrolment, a questionnaire will be completed including demographic information, medical history, treatment history, history of recent blood transfusions, and a thorough physical examination will be performed. A venous blood sample will be taken for CBC and LFT. Dried blood spot (DBS) will be made and will be used for subsequent parasite analyses. All participants will receive the second dose of chloroquine under supervision. Patients in both the arms will receive their first dose of primaquine.

Day 1: All participants will be reviewed and the final dose of chloroquine treatment will be provided. Patients in both study arms will receive their second dose of primaquine. Finger prick blood sample will be collected for haemoglobin measurement and microscopy. Physical examination of urine will be conducted.

Day 2: All participants will be reviewed and patients in both arms will receive their third dose of primaquine treatment under observation. Finger prick blood sample will be collected for haemoglobin measurement and microscopy. Physical examination of urine will be conducted.

Days 3–6: Participants in both arms will be treated with primaquine. Home visits will be conducted on alternate days and primaquine doses will be given for that day and the following day for both arms.

Day 7: The study team will visit the participant at home. A brief physical examination will be conducted by a Study Nurse. Finger prick blood sample will be collected for haemoglobin measurement and microscopy. Physical examination of urine will be conducted.

Days 8-13: Participants in the control arm will be treated with primaquine. Home visits will be conducted on alternate days and primaquine doses will be given for that day and the following day for the control arm. No interaction with patients in the intervention arm.

Days 14, 21, and 28 and months 2, 3, 4, 5, and 6: The study team will visit participants at home where they will be reviewed. A brief physical examination will be conducted by the Study Nurse. Finger prick blood sample will be collected for haemoglobin measurement and microscopy.

Unscheduled Visit: All participants with fever or symptoms indicative of malaria will be asked to return to the same health centre as on enrolment. A symptom questionnaire will be completed, a brief physical examination undertaken, and a venous blood sample will be collected and a malaria smear prepared for malaria diagnoses (read immediately) and CBC including haemoglobin and LFT measured. Physical examination of urine will be conducted. In patients with recurrent parasitaemia, DBS will be made and stored for parasite analysis.

Demographics: The patient's date of birth (or if not known, the estimated age) and gender will be recorded. For screening purposes and if the patient is a female of childbearing age, she will be asked if she is currently pregnant, lactating, planning to get pregnant and the date of the first day of her last menstrual period.

Medical history and physical examination: The details of any disease/surgical conditions, and drug allergies will be recorded. A thorough physical examination will be carried out. The patient's blood pressure, pulse rate, respiratory rate, height, weight and the results of a baseline physical examination will be recorded. The patient will be checked for pallor and jaundice. This will be done on screening and each subsequent follow-up visit.

Body weight: Body weight will be recorded on day 0/enrolment to the nearest kilogramme (Omron Digital weighing scale). The scales will be calibrated before use. Patients should wear minimal clothing while weighing to avoid overestimation. The screening weight will be used to satisfy the inclusion or exclusion criteria for nutritional status as well as to calculate the dose (number of tablets) to be administered.

Body temperature: Axillary temperature will be checked with a thermometer (Hicks digital thermometer)

having a precision of 0.1 °C. Temperature will also be measured as clinically indicated. If the result is < 36.0 °C, the reading will be repeated. Axillary temperature recording will be used throughout the study.

Plans to promote participant retention and complete follow-up {18b}

All patients will be counselled before enrolment about the requirements during the trial, in particular about the frequency of follow-up visits. The importance of completing the follow-up will be highlighted, but patients will also be reassured that they can drop out of the study at any time without providing a reason. In addition, participants will be compensated for wage loss and their travel costs.

Data management {19}

ICMR–National Institute of Malaria research will be responsible for data collection, storage, protection, retention and destruction. All documents will be stored securely and be accessible to trial staff and authorised personnel only. All study data will be recorded on standard Case Report Forms (CRF) and entered into a REDCap database, a GCP-compliant data management system. The database is password-protected and includes internal quality checks to identify data that appear inconsistent, incomplete, or inaccurate. Study participants will be identified by a unique participant number in the database. The study data management plan outlines all activities that will be carried out to ensure security and quality of the data. Subject records at the site will, taking into account the ability of the sites, be stored in binders or scanned and stored electronically. All records will be retained for 5 years following completion of the trial.

Confidentiality {27}

Study participants will be identified by a unique participant number in the CRF and the electronic database. The trial staff will ensure that the participants' anonymity is maintained. All documents and data will be stored securely and be accessible to trial staff and authorised personnel only. Only deidentified and anonymised specimens and data will be shared with other research groups.

Plans for collection, laboratory evaluation and storage of biological specimens for genetic or molecular analysis in this trial/future use {33}

The distinction between relapse and reinfection is an important parameter to understand the epidemiology of *P. vivax* malaria and the assessment of treatment efficacy. The cases of recurrence will be classified as relapse or reinfection based on the two genotyping methods PCR and sequencing. The method of PCR-RFLP will be done using *P. vivax*-specific highly polymorphic microsatellite markers and genes such as *Pv*MSP3α, *Pv*MSP4, *Pv*MS16, and *Pv*3.27 dried blood spot (DBS) will be made of all the enrolled patients. Parasite DNA will be extracted from DBS for PCR/Genotyping analysis. PCR/genotyping will be undertaken for speciation and genotyping before study drug treatment and in case of days of recurrence.

Sample collection

Three to 5 ml of EDTA-anticoagulated venous blood sample will be collected on screening (Day − 1) for malaria RDT & microscopy, G6PD testing (UV and Biosensor), dengue NS1 RDT and on enrolment (Day 0) for CBC including Hb estimation, and liver function tests. A venous blood sample will be collected on day of recurrence for CBC and LFT.

Finger prick blood sample will be collected for microscopy and haemoglobin estimation on enrolment and on each subsequent follow-up visit including the first day of each subsequent recurrence.

Microscopic blood examination

Specimens will be labelled anonymously (screening number or study number, day of follow-up, date). A fresh Giemsa stain dilution will be prepared at least once a day and possibly more often, depending on the number of slides to be processed. Giemsa-stained thick and thin blood films will be examined at a magnification of 1000× to identify the parasite species and to determine the parasite density.

Two blood slides per patient will be obtained: Both thick and thin blood smear on the same slide. One slide will then be stained rapidly (10% Giemsa for 10–15 min) for initial screening by confirming the presence or absence using the thin smear, while the second slide will be retained. If the patient is subsequently enrolled, the second slide will be stained more carefully (e.g. 6% Giemsa for 45–60 min), and slower staining will also be used for all slides obtained at follow-up visits. The study number of the patient, the date and the day of follow-up will be recorded either on the frosted edge of the slide or on the glass with a permanent glass pen.

On the slow stained slides, thick blood smear for initial screening will be used to count the numbers of asexual parasites as well as sexual (gametocytes) parasites. Thin smear will be used for species identification and parasite stage determination.

The second thick blood smear will be used to calculate the peripheral parasite density, by counting the number of asexual parasites per 500 white blood cells with a hand tally counter. Parasite density, expressed as the number of asexual parasites per µl of blood, will be calculated by dividing

the number of asexual parasites by the number of white blood cells counted and then multiplying by the actual white blood cell density (from the CBC reports on Day 0 for enrolled patients).

$$\text{Parasite density per } \mu\text{l} = \frac{\text{Number of parasites counted} \times \text{Actual WBC density}}{\text{Number of leukocytes counted}}$$

The same technique will be used to quantify parasite density on each subsequent blood film. A blood slide will be considered negative when examination of 200 high power fields (HPFs) reveals no asexual parasites.

In addition, 100 fields of the second thick film will be examined to exclude mixed infections; in case of any doubt, the thin film will be examined for confirmation. If examination of the thin film is not conclusive, the patient will be excluded from the analysis after complete treatment and follow-up.

Two qualified microscopists will read all the slides independently, and parasite densities will be calculated by averaging the two counts. Blood smears with discordant results (differences between the two microscopists in species diagnosis, in parasite density of > 50% or in the presence of parasites) will be re-examined by a third, independent microscopist, and parasite density will be calculated by averaging the two closest counts. This will be done on days − 1, 0, 1, 2, 7, 14, 21, and 28 and monthly until month 6 and the day of recurrence.

CBC and LFT

CBC and LFT will be measured on day 0 and day of recurrence, and repeated in between if the patient reports symptoms (jaundice, pallor) that warrant further investigation. Liver function parameters including total bilirubin, direct bilirubin, alkaline phosphatase, AST/ALT, total proteins, and albumin will be analysed. The tests and all the procedures will be outsourced as per the study protocol.

Heamoglobin

Haemoglobin will be measured using a point of care using a Hemocue™, which will be used according to manufacturer instructions. This will be done on days 0, 1, 2, 7, 14, 21, and 28 and monthly till month 6 and the day of recurrence.

G6PD deficiency testing

Prior to the study, the local Adjusted Male Median (AMM) will be calculated. G6PD activity will be measured quantitatively by UV spectrophotometer and SD Biosensor both on the day of enrolment. The results of the optimal G6PD activity by spectrophotometer will guide enrolment.

Dengue NS1 antigen test

The patients will be screened for dengue co-infection at the time of enrolment and during the next 14 days if patients turned febrile again.

For protocol-mandated blood tests that cannot be done immediately, DBS will be made on Day 0 and Day of recurrence for PCR/ genotyping. Any additional tests not listed in the protocol will need ethical approval.

Urine pregnancy test

Female patients of child-bearing age will be asked to take a urine ß HCG pregnancy test before enrolment in the study, because primaquine is contraindicated during pregnancy. Female participants of child-bearing age, defined as those who menstruate or are aged over 12 years, and who are sexually active should use oral contraceptives or barrier contraceptive devices (if oral contraceptives are contraindicated), or using an intrauterine device (IUD) for the duration of the study. Appropriate contraceptive methods will be suggested by the investigator or study team at the time informed consent will be obtained, with appropriate counselling about the risks of becoming pregnant and exposing the foetus to the study medicines.

Statistical methods

Statistical methods for primary and secondary outcomes {20a}

Efficacy analyses

The efficacy analyses will be based on the modified intention to treat population. Time to first recurrent vivax parasitaemia will be used to compute the Kaplan-Meier estimates of risk of *P. vivax* recurrence at month 6 for treatment arms and control arms with primaquine for each study site. Also, the comparison of the relative hazards between trial arms (hazard ratio (95% confidence interval)) will be estimated from a Cox regression model for the time to the first recurrent episode.

Safety analysis

All safety endpoints will be based on the safety population and presented in tabular and/or graphical format and summarised descriptively.

Clinical laboratory data (clinical chemistry and haematology) will be summarised by the mean, median, standard deviation, minimum and maximum values by treatment group and time point. Laboratory data will also be evaluated by tabulating the number and percentage of subjects in each treatment group with values outside specified threshold values of clinical concern (these may include values outside of the normal range, outer range of clinical concern, and other values of clinical concern).

AE reporting will be performed using the MedDRA (Medical Dictionaries for Regulatory Activities) coding system. Each AE coded using the MedDRA system can be associated with more than one system organ class (SOC). However, for reporting purposes, an AE will be associated with the primary system organ class only. Counting of AEs will be based on the number of subjects — not the number of AEs. For example, if a subject reports the same AE on three occasions within a time interval, that AE will only be counted only once. Subjects reporting more than one AE in a system organ class will only be counted once in that system organ class total. AEs considered by the investigator to have a reasonable possibility of being related to treatment (drug-related AEs) will be summarised by preferred term and SOC. AEs leading to premature withdrawal from treatment and or study will be summarised by preferred term and SOC. AEs that are considered to be gastro-intestinal-related (i.e abdominal pain, heartburn, diarrhoea, constipation, nausea and vomiting) will be summarised. AEs that are considered to be haematologically related (i.e clinically relevant drops in haemoglobin or haematocrit or other complications) will be summarised. Serious adverse events will be summarised by preferred term and SOC. All safety data relating to vital sign variables will be presented as summary statistics (including number, mean, median, interquartile range, standard deviation, minimum and maximum).

Interim analyses {21b}

No formal interim analysis has been planned, unless otherwise advised by the Data Safety Monitoring Board (DSMB).

Methods for additional analyses (e.g. subgroup analyses) {20b}

No subgroup analyses have been planned.

Methods in analysis to handle protocol non-adherence and any statistical methods to handle missing data {20c}

Patients can have an incomplete course of treatment or data on drug administration may be missing. No imputation of treatment courses will be made for patients with missing data.

Plans to give access to the full protocol, participant-level data and statistical code {31c}

The dataset will be uploaded on to WWARN and NIMR data server repositories.

Oversight and monitoring

Composition of the coordinating centre and trial steering committee {5d}

This is a study coordinated by the ICMR-National Institute of Malaria Research, New Delhi, India with study sites in Kolkata, West Bengal, India and Ahmedabad, Gujarat, India. Study Monitors will be appointed for each site. The monitor will verify the best conduct of the study through frequent contacts by phone and in person with the Principal Investigator and site staff, in accordance with the Standard Operating Procedures and Good Clinical Practice, with the purposes of facilitating the work and obtaining the objectives of the study. These visits will enable the monitor to maintain current, personal knowledge of the study through review of the records, comparison with source documents, observation, and discussion of the conduct of the study with the investigator.

Composition of the data monitoring committee, its role and reporting structure {21a}

A DSMB will be appointed for this study prior to its initiation. It will have 5-6 members, including a physician, subject experts and a biostatistician. It is responsible for safeguarding the interests of study participants, assessing predominantly the safety of study procedures, and for monitoring the overall conduct of the study.

Adverse event reporting and harms {22}

The investigator or site staff will be responsible for detecting, documenting and reporting events that meet the definition of adverse events (AEs) or serious adverse events (SAE), and any occurring events will be recorded on the CRF.

Definition of AE

Any untoward medical occurrence in a patient or clinical investigation subject, temporally associated with the use of a medicinal product, whether or not considered related to the medicinal product. For marketed medicinal products, this also includes failure to produce expected benefits (i.e. lack of efficacy), abuse or misuse.

The severity of an adverse event is to be scored according to the following scale:

- Mild: Awareness of sign or symptom, but easily tolerated
- Moderate: Discomfort enough to cause interference with usual activity

- Severe: Incapacitating with inability to work or perform usual activity

The relationship of an adverse event to study drug is to be assessed according to the following definitions:

- Definitely unrelated: Should be reserved for those events which occur prior to test drug administration (e.g. washout or single-blind placebo) or for those events which cannot be even remotely related to study participation (e.g. injury caused by a third party).
- Unlikely: There is no reasonable temporal association between the study drug and the suspected event and the event could have been produced by the subject's clinical state or other modes of therapy administered to the subject.
- Possible: The suspected adverse event may or may not follow a reasonable temporal sequence from study drug administration but seems to be the type of reaction that cannot be dismissed as unlikely. The event could have been produced or mimicked by the subject's clinical state or by other modes of therapy concomitantly administered to the subject.
- Probable: The suspected adverse event follows a reasonable temporal sequence from study drug administration, abates upon discontinuation of the drug, and cannot be reasonably explained by the known characteristics of the subject's clinical state.
- Definitely related: Should be reserved for those events which have no uncertainty in their relationship to test drug administration: this means that a rechallenge was positive.

The outcome of each AE must be assessed according to the following classification:

- Completely recovered: The patient has fully recovered with no observable residual effects
- Not yet completely recovered: Improvement in the patient's condition has occurred, but the patient still has some residual effects
- Deterioration: The patient's overall condition has worsened
- Permanent damage: The AE has resulted in a permanent impairment
- Death: The patient died due to the AE
- Ongoing: The AE has not resolved and remains the same as at onset
- Unknown: The outcome of the AE is not known because the patient did not return for follow-up (lost to follow-up)

Definition of SAE

A serious adverse event (SAE) is any untoward medical occurrence that, at any dose:

- Results in death
- Is life-threatening
- Requires hospitalisation or prolongation of existing hospitalisation
- Results in disability/incapacity
- Is a congenital anomaly/birth defect
- Requires intervention to prevent permanent impairment or damage

In the event of an SAE, the site investigator will report the case to the sponsor and chairman of the ethics committee (EC) within 24 h of its occurrence. The report of SAE will then be forwarded by the principal investigator to chairman of EC within 14 days of its occurrence. Trial participants who suffer direct physical, psychological, social, legal or economic harm as a result of participating in the trial are entitled to free health care and referrals as needed. Where any death of a participant occurs during the clinical trial, the legal heir of the trial subject shall be provided financial compensation by the National Institute of Malaria Research (NIMR-New Delhi) through insurance coverage.

The EC is responsible for reviewing the relatedness of the SAE to the research, as reported by the researcher, and determining the quantum and type of assistance to be provided to the participants. All research participants who suffer harm, whether related or not, should be offered appropriate medical care, psycho-social support, referrals, clinical facilities, etc. Medical management should be free if the harm is related to the research. Compensation should be given to any participant when the injury is related to the research. This is applicable to participants in any of the arms of research, such as intervention, control and standard of care. The quantum of compensation to be awarded to participants who have suffered research-related injury, the EC will consider aspects including the type of research (interventional, observational, etc.), extent of injury (temporary/permanent, short/long term), loss of wages, etc.

All the patients enrolled in the study will be reimbursed for their daily wage loss and transportation.

Frequency and plans for auditing trial conduct {23}

A detailed monitoring plan is prepared. In brief, this will state that a minimum of four in-person monitoring visits will be conducted during the course of the study.

Monitoring frequency may be increased based on the following criteria among others: (i) high subject enrolment at a particular site; (ii) compliance issues (e.g.

significant protocol violations); (iii) site staff turnover, requiring additional training; (iv) results and findings from previous monitoring visit.

Auditing can also take place by national health authorities at the participating study sites.

Plans for communicating important protocol amendments to relevant parties (e.g. trial participants, ethical committees) {25}

All protocol modifications will be communicated promptly to all involved parties and updated in the clinical trials registry. Any protocol deviations will be fully documented.

Dissemination policy {31a}

Results will be published in peer-reviewed journals. All investigators will be involved in reviewing drafts of the manuscripts, abstracts, press releases, and any other publications arising from the study. Authorship will be determined in accordance with the ICMJE guidelines and other contributors will be acknowledged. Results will further be disseminated at international conferences and other stakeholder meetings including the National Centre for Vector Borne Disease Control in India. All efforts will be made to disseminate results to study participants in line with local requirements.

Discussion

India aims to eliminate malaria by 2030. One of the major challenges to achieve this goal is the radical cure of patients with *P. vivax* malaria. The latter can be undermined by long treatment courses, resulting in poor adherence and reduced effectiveness. Shorter treatment options have now been recommended by WHO[7] but additional local evidence of their efficacy and safety are required for the Indian malaria program to inform implementation and change policy. Earlier studies [11, 12, 14] did not generate enough evidence for policy change. This study will directly inform treatment policy.

Trial status

Started

Protocol number: NIMR-IDDO/PQ/2022-01

Version number and date: Version 03, Date-14/03/2023

Recruitment start date: 11/9/23

Estimated date of completion of recruitment: Mar. 2024

Acknowledgements

We thank all the project staffs in this clinical trial at both the sites, and the WWARN team for technical and administrative support.

Authors' contributions {31b}

Study Conception: AS, KT, MR, PJG, RJC, RNP, Funding acquisition: PJG. Project administration: N, PKB, SSP. Resources: MR, N, PKB, PJG. Writing—original draft: RV, SSA. Writing—review and editing: All authors critically edited, read and approved the initial and final versions.

Availability of data and materials {29}

Study Protocol and Statistical Analysis Plan will be made available to others. Data collected for the study, including individual patient data and the final trial dataset, are reserved for the principal investigator and co-investigators of the trial. Trial results will be published in peer-reviewed open access journals and disseminated to trial stakeholders, including participants, as per ethical guidelines.

Funding {4}

Infectious Diseases Data Observatory, University of Oxford, New Richards Building, Old Road Campus, Roosevelt Drive, Oxford, OX3 7LG, UK through a grant from the Bill and Melinda Gates Foundation to PJG (grant number: INV-004713). The funders had no role in the design or conduct of the study.

Declarations

Ethics approval and consent to participate {24}

ICMR- National Institute of Malaria Research has obtained approval from the Institutional Ethics Committee (IEC) with relevant documents after Scientific Advisory Committee (SAC) approval. Ethics approval has been obtained from both site ethics committees as well. The proposal has also been submitted for the Health Ministry Screening Committee's (HMSC) approval. The trial has been registered online prospectively through the Clinical Trial Registry of India (CTRI) after obtaining the approval of the IEC and receiving exemption from the Drug Controller General of India. The study will be conducted in accordance with Good Clinical Practice (GCP), all applicable subject privacy requirements, and the ethical principles that are outlined in the Declaration of Helsinki 2008.

Consent for publication {32}

This manuscript does not contain individual personal data from patients they will be presented in reports of the trial results. Informed consent materials are available, on request, from the corresponding author.

Competing interests {28}

The authors declare they have no competing interests.

Author details

[1]ICMR-National Institute of Malaria Research, New Delhi, India. [2]WorldWide Antimalarial Resistance Network, Asia-Pacific Regional Hub, Melbourne, Australia. [3]Menzies School of Health Research and Charles Darwin University, Darwin, Australia. [4]General and Subspecialty Medicine, Grampians Health, Ballarat, Australia. [5]Infectious Diseases Data Observatory – IDDO, Oxford, UK. [6]Centre for Tropical Medicine and Global Health, Nuffield Department of Medicine, University of Oxford, New Richards Building, Old Road Campus, Roosevelt Drive, Oxford OX3 7LG, UK. [7]Delhi Skill and Entrepreneurship University, New Delhi, India. [8]Calcutta National Medical College, Kolkata, West Bengal, India. [9]NIMR Field Unit, Academy of Scientific and Innovative Research, Ghaziabad, Gujarat, India. [10]Ahmedabad Municipal Corporation, Ahmedabad, Gujarat, India. [11]BJ Medical College, Ahmedabad, Gujarat, India. [12]International Centre for Genetic Engineering and Biotechnology, New Delhi, India. [13]Mahidol-Oxford Tropical Medicine Research Unit, Faculty of Tropical Medicine, Mahidol University, Bangkok, Thailand. [14]Academy of Scientific and Innovative Research (AcSIR), Ghaziabad, India. [15]Indian Council of Medical Research, New Delhi, India.

Received: 24 November 2023 Accepted: 15 February 2024
Published online: 29 February 2024

References

1. World Malaria Report 2022. Available on https://www.who.int/teams/global-malaria-programme/reports/world-malaria-report-2022. Accessed 21 Mar 2023.
2. National Centre for Vector Borne Disease Control. Available at: https://ncvbdc.mohfw.gov.in/index.php Accessed 22 Mar 2023.
3. Plasmodium vivax in the era of the shrinking P. falciparum map. 10.1016/j.pt.2020.03.009
4. Fernando D, Rodrigo C, Rajapakse S. Primaquine in vivax malaria: an update and review on management issues. Malaria J. 2011;10(1):1–12.
5. Pal S, Myburgh J, Bansil P, et al. Reference and point-of-care testing for G6PD deficiency: Blood disorder interference, contrived specimens, and fingerstick equivalence and precision. PLoS One. 2021;16(9):e0257560. https://doi.org/10.1371/journal.pone.0257560.
6. Guidelines for diagnosis and treatment of Malaria 2014. National Centre for Vector Borne Disease Control. Available at: https://nvbdcp.gov.in/WriteReadData/l892s/20627628441542176662.pdf Accessed 21 Mar 2023.
7. Dhariwal AC, Sonal GS, Kumar A, et al. Guidelines on diagnosis and treatment of malaria in India. Third. J Indian Med Assoc. 2015;113:207–10.
8. Douglas NM, Poespoprodjo JR, Patriani D, et al. Unsupervised primaquine for the treatment of Plasmodium vivax malaria relapses in southern Papua: A hospital-based cohort study. PLoS Med. 2017;14(8):e1002379. https://doi.org/10.1371/journal.pmed.1002379.
9. Rahi M, Sirohi PR, Sharma A. Supervised administration of primaquine may enhance adherence to radical cure for P. vivax malaria in India. Lancet Reg Health Southeast Asia. 2023;13:100199. https://doi.org/10.1016/j.lansea.2023.100199.
10. WHO Guidelines for Malaria November 2022. Available on https://www.who.int/publications/i/item/guidelines-for-malaria Accessed 18 Dec 2022.
11. Pareek A, et al. Sustained release formulation of primaquine for prevention of relapse of Plasmodium vivax malaria: a randomized, double-blind, comparative, multicentric study. Malar Res Treat. 2015;2015:579864.
12. Rajgor DD, Gogtay NJ, Kadam VS, et al. Antirelapse Efficacy of various primaquine regimens for Plasmodium vivax. Malar Res Treat. 2014;2014:347018. https://doi.org/10.1155/2014/347018.
13. Verma R, Commons RJ, Gupta A, Rahi M, Nitika, Bharti PK, Thriemer K, Rajasekhar M, Singh-Phulgenda S, Adhikari B, Alam MS, Ghimire P, Khan WA, Kumar R, Leslie T, Ley B, Llanos-Cuentas A, Pukrittayakamee S, Rijal KR, Rowland M, Saravu K, Simpson JA, Guerin PJ, Price RN, Sharma A. Safety and efficacy of primaquine in patients with Plasmodium vivax malaria from South Asia: a systematic review and individual patient data meta-analysis. BMJ Glob Health. 2023;8(12):e012675. https://doi.org/10.1136/bmjgh-2023-012675.
14. Rajgor DD, Gogtay NJ, Kadam VS, et al. Efficacy of a 14-day primaquine regimen in preventing relapses in patients with *Plasmodium vivax* malaria in Mumbai, India. Trans R Soc Trop Med Hyg. 2003;97(4):438–40.

Publisher's Note

Springer Nature remains neutral with regard to jurisdictional claims in published maps and institutional affiliations.
