# Supplementary material for: Seven-day versus fourteen-day low-dose primaquine for Plasmodium vivax malaria in India: an open-label, non-inferiority, randomised, controlled trial
Source: Lancet Reg Health Southeast Asia. 2026 Aug 25;53:100849. doi: 10.1016/j.lansea.2026.100849 (PMC13651684; doi:10.1016/j.lansea.2026.100849)
Supplement: Supplementary file 2 [file mmc2.pdf]

Statistical Analysis Plan

A multi-center randomized controlled trial to compare the efficacy, safety, and tolerability of high dose, short course Primaquine

Version number: 2

Date: 1st April 2025

Contents

1. Introduction 3

2. Study design and objectives 3

2.1. Summary of trial design 3

2.2. Objectives 4

Primary Objective 4

2.3. Sample size 4

3. Endpoints 4

3.4 Handling of missing data and adjudication of Endpoints 6

3.4.1 Handling of missing data on drug course 6

3.4.2 Handing of missing data for Adverse Events 6

3.4.3 Adjudication of efficacy outcome assessment 6

3.5 Analyses Populations 7

4 Analyses 8

4.1 Demographic and baseline characteristics 8

4.1.1 Demographic characteristics 8

4.1.2 Disease characteristics at baseline 8

4.2 Efficacy analyses 9

4.2.1 Incidence risk of symptomatic P. vivax parasitaemia at month 6 (180 days) 9

4.2.2 The incidence risk of any P. vivax parasitaemia at month 6 9

4.3 Safety analyses 10

4.3.1 Proportion of patients vomiting their medication within 1 hour of administration 10

4.3.2 Proportion of adverse events and serious adverse events 10

4.3.4 Proportion of patients with greater than 25% fall in haemoglobin on any day of treatment since baseline 10

4.3.5 Proportion of patients with acute drop in Hb of >5g/dl during PQ treatment 10

1. Introduction

This Statistical Analysis Plan (SAP) provides a detailed and comprehensive description of the main pre-planned analyses for the clinical trial " A multi-center randomized controlled trial to compare the efficacy, safety, and tolerability of high dose, short course Primaquine".

The main purpose of the trial is to compare the efficacy and safety of a total dose of 3.5 mg/kg primaquine (PQ) in a 7-day regimen provided as 0.50 mg/kg/day with the standard treatment regimen of 0.25 mg/kg/day for 14 days, with a follow-up period up to 6 months. The study conduct is described in Protocol - NIMR-IDDO/PQ/2022-01 and registered with CTRI under CTRI/2022/12/048283. This document describes the statistical methods for the primary and secondary outcomes of the study as defined by the protocol.

2. Study design and objectives

2.1. Summary of trial design

This is an open label, parallel group, multicentre randomised trial with a six month follow up post treatment. There are two arms, with one arm receiving PQ for 14 days prescribed as per national guidelines (0.25 mg/kg/day) and the other group receiving PQ for 7 days (0.50 mg/kg/day).

<u>Intervention arm</u>:

PQ 0.50 mg/kg/day for 7 days

<u>Control arm</u>:

PQ 0.25 mg/kg/day for 14 days

Patients will be tested for G6PD activity and those found to be G6PD deficient (<30% of adjusted male median value) will be excluded from the study. (The complete inclusion and exclusion criteria are provided in the protocol).

PQ will be administered once per day with food (crackers or a biscuit) to reduce gastrointestinal side effects. Alternate doses of PQ will be supervised. If participants cannot visit the study centre, or fail to attend, team members will visit them in their homes to encourage complete dosing and for blood sampling. Treatment efficacy and patient safety will be ensured by close monitoring over 6 months; following a schedule of visits and corresponding clinical and laboratory examinations.

2.2. Objectives

Primary Objective

- To assess the efficacy of a 7-day course of PQ given as 0.50 mg/kg/day (3.5 mg/kg total dose) in preventing recurrent symptomatic *P. vivax* parasitaemia in G6PD normal patients following uncomplicated *P. vivax* infection compared to the current standard regimen of 0.25 mg/kg/day PQ given over 14 days (3.5 mg/kg total dose).
- To assess the safety of a 7-day course of PQ (0.50 mg/kg/day) compared to the current standard PQ regimen given over 14 days (0.25 mg/kg/day).

2.3. Sample size

The 0.25 mg/kg/day for 14 days regimen of PQ is associated with an approximate risk of vivax recurrence of 13% over 6 months. The present study will assess if 0.5 mg/kg/day for 7 day regimen of PQ will have a non-inferior recurrence rate within a 10% non-inferiority margin. A sample size of 200 per group, assuming a 30% loss to follow-up at a one-sided significance level of 0.025 will provide 68% power to detect this difference. Accordingly, we aim to enrol 200 patients at each site (100 in each arm), 400 in total.

3. Endpoints

3.1 Primary efficacy endpoints

Efficacy related outcome

- The cumulative incidence of symptomatic *P. vivax* malaria till month 6.

3.2 Safety endpoints

- The proportion of patients vomiting their medication within 1 hour of administration
- The proportion of adverse events within 28 days on initial treatment
- The proportion of serious adverse events within 6 months on initial treatment
- The proportion of patients with severe anaemia (Hb<8g/dL) and/or the risk for blood transfusion on days 1-14
- Risk of greater than 25% fall in haemoglobin from day 0 to days 1-14
- The proportion of patients with an acute drop in Hb of >5g/dL from day 0 to days 1-14.
- Gastrointestinal (GI) tolerability - proportion of patients with abdominal pain, heartburn, diarrhoea, constipation, nausea and vomiting on Day 2, 7 and 14

3.3 Secondary efficacy endpoints

The cumulative incidence of any (symptomatic and asymptomatic) *P. vivax* malaria at month 6.

3.4 Handling of missing data and adjudication of Endpoints

3.4.1 Handling of missing data on drug course

Patients can have an incomplete course of treatment or data on drug administration may be missing. No imputation of treatment course will be made for patients with missing data. Subgroup analysis will be performed for patients with full versus partial treatment courses.

3.4.2 Handing of missing data for Adverse Events

For patients with missing data on adverse events the most conservative approach will be used:

Deviation	Action
Start date of AE missing	Assume during study drug intake
End date of AE missing	No imputation
Date of start of study treatment administration missing	Enrollment date to be considered as the start of study treatment
Missing assessment of relationship to study treatment	Assume event to be possibly related
Missing severity assessment of AE	Assume highest severity

3.4.3 Adjudication of efficacy outcome assessment

The clinical definition of treatment failure enables determination of the efficacy endpoint for antimalarial activity. However, when the number of scheduled visits is incomplete the following rules will be applied:

Deviation	Action
More than 2 consecutive months without blood film examination.	In the survival analysis lost to follow up (i.e. censored) on the day of last visit before missing observation period.

3.5 Analyses Populations

3.5.1 Intention to Treat

To provide a pragmatic comparison of the different drug treatments, the principle of intention-to-treat, will be considered.

Efficacy analyses will be conducted on the intention-to-treat (ITT) population, encompassing all randomized participants analyzed according to their originally assigned treatment groups, regardless of adherence to the protocol or treatment received.

Time to first recurrent vivax parasitaemia will be used to compute the Kaplan-Meier estimates of risk of the first *P. vivax* recurrence during 6 months for both the intervention and control PQ arms.

3.5.2 Per Protocol

An analysis based on a "per protocol" approach will be conducted following the causal framework presented in Hernan & Robins[1]. The following participants will be excluded from the per protocol analyses:

- Ineligibility (either arising during the study or retrospectively having been overlooked at screening)
- Significant protocol deviation (e.g., given wrong total dose of primaquine)
- Incomplete treatment course

[1] Hernan & Robins. Per-protocol Analyses of Pragmatic Trials. N Engl J Med. 2017 Oct 5;377(14):1391-1398. doi: 10.1056/NEJMsm1605385.

3.5.3 Safety population

For the analysis of safety outcomes, all patients who received any study drug are included in the safety analysis in the treatment group they actually received (i.e. that they were administered). This means that patients having vomited their first dose or having had their treatment discontinued will be included in this analysis. All safety endpoints will be based on the safety population and presented in tabular and/or graphical format and summarised descriptively.

4 Analyses

4.1 Demographic and baseline characteristics

Details of all patients screened, those who meet the study inclusion criteria, those who are eligible and randomized, those who are eligible but not randomized, those who withdraw from the study after randomization and those who are lost to follow-up will be summarized in a CONSORT flow diagram.

The number of patients discontinuing from the study will be tabulated by reason for study discontinuation. The number (%) of patients attending/completed scheduled follow-up visits by study day will be reported. The baseline value is defined as the last available value before randomization.

The following demographic and disease characteristics at baseline by treatment group will be presented.

4.1.1 Demographic characteristics

- Gender: male / female
- Median Age (years), 25th-75th percentiles, and range
- Median Weight (kg), 25th-75th percentiles, and range
- Weight in classes: <45 kg, 45-60 kg, > 60 kg
- Trial site

4.1.2 Disease characteristics at baseline

Specific disease history will include the parasite densities (/µL) at day 0, as well as axillary temperature (°C) (quantitative and if <37.5°C), and pulse rate (beats/minute), presented as median, 25th-75th percentiles, and range. The frequency and percent of symptoms (fever, chills malaise, fatigue, muscle pain, joint pain, headache, irritability, nausea, vomiting, diarrhea, abdominal pain, loss of appetite, jaundice, shortness of breath, dizziness, dark urine), and haemoglobin at day 0 will be presented.

4.2 Efficacy analyses

4.2.1 Incidence risk of symptomatic *P. vivax* parasitaemia at month 6 (180 days)

The incidence risk (95% CI) of parasite recurrence within 6 months of follow-up will be calculated using the Kaplan-Meier (KM) method for each trial arm as well as a comparison of the relative hazards between trial arms. Hazard Ratio (95% CI) will be estimated using a Cox regression model for the time to the first recurrent episode. The **first recurrent symptomatic episode** of *P. vivax* will be treated as the failure endpoint. Patients presenting with symptomatic parasitaemia other than *P. vivax* will be censored at the day of occurrence. Patients with asymptomatic parasitaemia will be censored at the time of occurrence.

The patients who are categorised as lost to follow up will be censored at the last visit day.

Comparisons will be made between the intervention and control arm. The analyses will be performed for the ITT and the PP population with stratification for site.

4.2.2 The incidence risk of any *P. vivax* parasitaemia at month 6

The first recurrent episode of *P. vivax* (symptomatic or asymptomatic) will be treated as a failure endpoint. Patients presenting with parasitaemia other than *P. vivax* will be censored at the day of occurrence.

This analysis will be performed as described in section 4.2.1. with follow up to month 6. Comparisons will be made between the intervention and control arm. The analyses will be performed for the ITT and the PP population.

4.3 Safety analyses

The primary safety concerns regarding primaquine use pertain to the risk of haemolysis and gastroenterological adverse events.

4.3.1 Proportion of patients vomiting their medication within 1 hour of administration

The proportion of patients with vomiting within 1 hour of administration of a dose of PQ will be calculated for each arm. The absolute difference between the intervention arm and the control arm will be presented and tested for statistical significance using the chi-square test.

4.3.2 Proportion of adverse events and serious adverse events

The proportion of patients with one or more adverse events (till day 28) and serious adverse events (within 6 months) will be presented. Events will be divided into related and unrelated events with respect to the treatment.

4.3.3 Proportion of patients with severe anaemia (Hb<8g/dl) and/or the risk for blood transfusion

The proportion of severe anaemia will be calculated using the Kaplan-Meier method. Patients who develop severe anaemia (defined as i) dropping <8g/dl; and/or ii) requiring blood transfusion) on any day of PQ treatment will be considered failures. Patients lost to follow up or with missing measurements will be censored at the time of the last visit.

4.3.4 Proportion of patients with greater than 25% fall in haemoglobin on any day of treatment since baseline

The proportion of greater than 25% fall in haemoglobin on any day of PQ treatment since baseline will be calculated using the Kaplan-Meier method. Patients who develop a drop in Hb >= 25% compared to baseline will be considered as failures/endpoint. Patients lost to follow up or with missing measurements will be censored at the time of the last visit.

4.3.5 Proportion of patients with acute drop in Hb of >5g/dl during PQ treatment

The proportion of patients with acute drop in Hb of >5g/dl will be calculated using the Kaplan-Meier method. Patients who develop a drop in >5g on any day of PQ treatment compared to baseline during will be considered failures. Patients lost to follow up or with missing measurements will be censored at the time of the last visit.

4.3.6 GI intolerability

The proportion of patients experiencing gastrointestinal symptoms—including abdominal pain, heartburn, diarrhea, constipation, nausea, and vomiting—will be assessed as a composite endpoint at Days 0, 2, 7, and 14 for each study arm. Comparative analyses between the intervention and control groups will be conducted using the chi-square test to evaluate statistical significance.
