# Supplementary material for: Seven-day versus fourteen-day low-dose primaquine for Plasmodium vivax malaria in India: an open-label, non-inferiority, randomised, controlled trial
Source: Lancet Reg Health Southeast Asia. 2026 Aug 25;53:100849. doi: 10.1016/j.lansea.2026.100849 (PMC13651684; doi:10.1016/j.lansea.2026.100849)
Supplement: Supplementary file 3 [file mmc3.pdf]

Supplementary file 3 – Appendix

Baharia RK, Singh-Phulgenda S, et al, *Seven-day versus fourteen-day low-dose primaquine for radical cure of Plasmodium vivax malaria in India: an open-label, non-inferiority, randomised, controlled trial*, 2026

Table of Contents

CHECKLIST S1: CONSORT 2025 CHECKLIST 2

CHECKLIST S2: CONSORT EXTENSION CHECKLIST FOR NON-INFERIORITY TRIALS 7

TABLE S1: DETAILS OF STUDY SITES 9

TABLE S2: BASELINE CHARACTERISTICS OF PATIENTS ENROLLED IN AHMEDABAD 10

TABLE S3: BASELINE CHARACTERISTICS OF PATIENTS ENROLLED IN KOLKATA 11

FIGURE S1: TRIAL PROFILE OF AHMEDABAD SITE 12

FIGURE S2: TRIAL PROFILE OF KOLKATA SITE 13

FIGURE S3: PRIMAQUINE MG/KG DOSE BY ARM 14

FIGURE S4: CUMULATIVE INCIDENCE OF ANY PARASITAEMIA BY DAY 180 15

FIGURE S5: CUMULATIVE INCIDENCE OF SYMPTOMATIC PARASITAEMIA BY DAY 180 AT A) AHMEDABAD AND B) KOLKATA 16

FIGURE S6: CUMULATIVE INCIDENCE OF SYMPTOMATIC PARASITAEMIA BY DAY 180 IF COMPLETE ADHERENCE TO PRIMAQUINE (PER PROTOCOL ANALYSIS) 17

FIGURE S7: CUMULATIVE INCIDENCE OF SYMPTOMATIC PARASITAEMIA BY DAY 180 FOR A) MALES AND B) FEMALES 18

Checklist S1: CONSORT 2025 Checklist

Section/topic	No	CONSORT 2025 checklist item description	Reported on page no.
**Title and abstract**			
Title and structured abstract	1a	Identification as a randomised trial	1
	1b	Structured summary of the trial design, methods, results, and conclusions	3-4
**Open science**			
Trial registration	2	Name of trial registry, identifying number (with URL) and date of registration	3
Protocol and statistical analysis plan	3	Where the trial protocol and statistical analysis plan can be accessed	Included in supplement
Data sharing	4	Where and how the individual de-identified participant data (including data dictionary), statistical code and any other materials can be accessed	20
Funding and conflicts of interest	5a	Sources of funding and other support (eg, supply of drugs), and role of funders in the design, conduct, analysis and reporting of the trial	21
	5b	Financial and other conflicts of interest of the manuscript authors	20-21
**Introduction**			
Background and rationale	6	Scientific background and rationale	7-8
Objectives	7	Specific objectives related to benefits and harms	8
**Methods**			

Patient and public involvement	8	Details of patient or public involvement in the design, conduct and reporting of the trial	N/A
Trial design	9	Description of trial design including type of trial (eg, parallel group, crossover), allocation ratio, and framework (eg, superiority, equivalence, non-inferiority, exploratory)	8-10
Changes to trial protocol	10	Important changes to the trial after it commenced including any outcomes or analyses that were not prespecified, with reason	NA
Trial setting	11	Settings (eg, community, hospital) and locations (eg, countries, sites) where the trial was conducted	9 and supplement
Eligibility criteria	12a	Eligibility criteria for participants	9
	12b	If applicable, eligibility criteria for sites and for individuals delivering the interventions (eg, surgeons, physiotherapists)	NA
Intervention and comparator	13	Intervention and comparator with sufficient details to allow replication. If relevant, where additional materials describing the intervention and comparator (eg, intervention manual) can be accessed	9-10 and supplement
Outcomes	14	Prespecified primary and secondary outcomes, including the specific measurement variable (eg, systolic blood pressure), analysis metric (eg, change from baseline, final value, time to event), method of aggregation (eg, median, proportion), and time point for each outcome	11-12
Harms	15	How harms were defined and assessed (eg, systematically, non-systematically)	12
Sample size	16a	How sample size was determined, including all assumptions supporting the sample size calculation	12

	16b	Explanation of any interim analyses and stopping guidelines	N/A
Randomisation:			
Sequence generation	17a	Who generated the random allocation sequence and the method used	9-10
	17b	Type of randomisation and details of any restriction (eg, stratification, blocking and block size)	9-10
Allocation concealment mechanism	18	Mechanism used to implement the random allocation sequence (eg, central computer/telephone; sequentially numbered, opaque, sealed containers), describing any steps to conceal the sequence until interventions were assigned	9-10
Implementation	19	Whether the personnel who enrolled and those who assigned participants to the interventions had access to the random allocation sequence	10
Blinding	20a	Who was blinded after assignment to interventions (eg, participants, care providers, outcome assessors, data analysts)	NA
	20b	If blinded, how blinding was achieved and description of the similarity of interventions	NA
Statistical methods	21a	Statistical methods used to compare groups for primary and secondary outcomes, including harms	12-13
	21b	Definition of who is included in each analysis (eg, all randomised participants), and in which group	12-13
	21c	How missing data were handled in the analysis	SAP in supplement
	21d	Methods for any additional analyses (eg, subgroup and sensitivity analyses), distinguishing prespecified from post hoc	12-13

**Results**			
Participant flow, including flow diagram	22a	For each group, the numbers of participants who were randomly assigned, received intended intervention, and were analysed for the primary outcome	Fig 1
	22b	For each group, losses and exclusions after randomisation, together with reasons	Fig 1
Recruitment	23a	Dates defining the periods of recruitment and follow-up for outcomes of benefits and harms	13
	23b	If relevant, why the trial ended or was stopped	NA
Intervention and comparator delivery	24a	Intervention and comparator as they were actually administered (eg, where appropriate, who delivered the intervention/comparator, how participants adhered, whether they were delivered as intended (fidelity))	NA
	24b	Concomitant care received during the trial for each group	NA
Baseline data	25	A table showing baseline demographic and clinical characteristics for each group	Table 1
Numbers analysed, outcomes and estimation	26	For each primary and secondary outcome, by group:<br>● the number of participants included in the analysis<br>● the number of participants with available data at the outcome time point<br>● result for each group, and the estimated effect size and its precision (such as 95% confidence interval)<br>● for binary outcomes, presentation of both absolute and relative effect size	14-15 and Tables 2-3 and Fig 2
Harms	27	All harms or unintended events in each group	14-15 and Tables 2-3

Ancillary analyses	28	Any other analyses performed, including subgroup and sensitivity analyses, distinguishing pre-specified from post hoc	N/A
**Discussion**			
Interpretation	29	Interpretation consistent with results, balancing benefits and harms, and considering other relevant evidence	16-19
Limitations	30	Trial limitations, addressing sources of potential bias, imprecision, generalisability, and, if relevant, multiplicity of analyses	18-19

Checklist S2: CONSORT extension checklist for non-inferiority trials

PAPER SECTION And topic	Item	Descriptor	Reported on Page #
TITLE & ABSTRACT	1	How participants were allocated to interventions (e.g., "random allocation", "randomized", or "randomly assigned"), specifying that the trial is a non-inferiority or equivalence trial.	3
INTRODUCTION Background	2	Scientific background and explanation of rationale, including the rationale for using a non-inferiority or equivalence design.	7-8
METHODS Participants	3	Eligibility criteria for participants (detailing whether participants in the non-inferiority or equivalence trial are similar to those in any trial(s) that established efficacy of the reference treatment) and the settings and locations where the data were collected.	9
Interventions	4	Precise details of the interventions intended for each group detailing whether the reference treatment in the non-inferiority or equivalence trial is identical (or very similar) to that in any trial(s) that established efficacy, and how and when they were actually administered.	11
Objectives	5	Specific objectives and hypotheses, including the hypothesis concerning non-inferiority or equivalence.	13
Outcomes	6	Clearly defined primary and secondary outcome measures detailing whether the outcomes in the non-inferiority or equivalence trial are identical (or very similar) to those in any trial(s) that established efficacy of the reference treatment and, when applicable, any methods used to enhance the quality of measurements (e.g., multiple observations, training of assessors).	12
Sample size	7	How sample size was determined detailing whether it was calculated using a non-inferiority or equivalence criterion and specifying the margin of equivalence with the rationale for its choice. When applicable, explanation of any interim analyses and stopping rules (and whether related to a non-inferiority or equivalence hypothesis).	13
Randomization -- Sequence generation	8	Method used to generate the random allocation sequence, including details of any restrictions (e.g., blocking, stratification)	10
Randomization -- Allocation concealment	9	Method used to implement the random allocation sequence (e.g., numbered containers or central telephone), clarifying whether the sequence was concealed until interventions were assigned.	10
Randomization -- Implementation	10	Who generated the allocation sequence, who enrolled participants, and who assigned participants to their groups.	10
Blinding (masking)	11	Whether or not participants, those administering the interventions, and those assessing the outcomes were	10

		blinded to group assignment. If done, how the success of blinding was evaluated.	
Statistical methods	12	Statistical methods used to compare groups for primary outcome(s), specifying whether a one or two-sided confidence interval approach was used. Methods for additional analyses, such as subgroup analyses and adjusted analyses.	14
RESULTS<br>Participant flow	13	Flow of participants through each stage (a diagram is strongly recommended). Specifically, for each group report the numbers of participants randomly assigned, receiving intended treatment, completing the study protocol, and analyzed for the primary outcome. Describe protocol deviations from study as planned, together with reasons.	Figure 1
Recruitment	14	Dates defining the periods of recruitment and follow-up.	15
Baseline data	15	Baseline demographic and clinical characteristics of each group.	Table 1
Numbers analyzed	16	Number of participants (denominator) in each group included in each analysis and whether the analysis was "intention-to-treat" and/or alternative analyses were conducted. State the results in absolute numbers when feasible (e.g., 10/20, not 50%).	15-16
Outcomes and estimation	17	For each primary and secondary outcome, a summary of results for each group, and the estimated effect size and its precision (e.g., 95% confidence interval). For the outcome(s) for which non-inferiority or equivalence is hypothesized, a figure showing confidence intervals and margins of equivalence may be useful.	15-16
Ancillary analyses	18	Address multiplicity by reporting any other analyses performed, including subgroup analyses and adjusted analyses, indicating those pre-specified and those exploratory.	15-16
Adverse events	19	All important adverse events or side effects in each intervention group.	16
DISCUSSION<br>Interpretation	20	Interpretation of the results, taking into account the non-inferiority or equivalence hypothesis and any other study hypotheses, sources of potential bias or imprecision and the dangers associated with multiplicity of analyses and outcomes.	17-21
Generalizability	21	Generalizability (external validity) of the trial findings.	20
Overall evidence	22	General interpretation of the results in the context of current evidence.	17-21

Table S1: Details of Study sites

	**Kolkata, West Bengal**	**Ahmedabad, Gujarat**
Geographic setting	Eastern India	Western India
Urban classification	Large metropolitan city; High population density, migrant labour populations, urban slums, seasonal mobility	Large metropolitan city; Mixed urban and peri-urban populations, industrial labour zones, migrant communities
Estimated population served	~14–15 million (Kolkata metropolitan area)	~8–9 million (Ahmedabad urban agglomeration)
Primary trial site & Recruitment network	Calcutta National Medical College (CNMC)<br>Patients recruited via Kolkata Municipal Corporation (KMC) health centres	Ahmedabad Municipal Corporation (AMC) health system.<br>Patients recruited from CHCs at Vatva, Vastral, Sarkhej, Rakhyal and LG General Hospital
P. vivax epidemiology	*P. vivax* is the dominant species of reported malaria cases in urban Kolkata, with transmission reported through municipal surveillance and outpatient case detection	*P. vivax* is the dominant species of reported malaria cases in Ahmedabad, particularly in peri-urban and industrial catchments

Table S2: Baseline characteristics of patients enrolled in Ahmedabad

	7 days PQ N=118	14 days PQ N=118
Age (years)	34.0 (24.0-47.0)	34.5 (24.0-42.0)
Sex		
Male	95/118 (80.5%)	97/118 (82%)
Female	23/118 (19.5%)	21/118 (18%)
Weight (kg)	58.0 (54.0-65.0)	58.0 (53.2-65.0)
Parasite density day -1 (/uL)	1283.6 (617.1-3403.8)	1323.6 (625.4-3478.2)
Body temperature day 0 (°C)	38.7 (38.2-39.2)	38.6 (38.3-39.2)
Presence of fever in the previous 48h	118/118 (100%)	118/118 (100%)
Haemoglobin day 0 (g/dL)	12.3 (1.8)	12.0 (1.9)
G6PD activity (U/gHb)	12.7 (11.5-14.9)	13.5 (11.4-15.6)
Primaquine mg/kg daily dose	0.5 (0.5-0.6)	0.3 (0.2-0.3)
Reported missed primaquine dose	5/118 (4%)	10/118 (8%)

Data are presented as mean (SD) or median (IQR) for continuous measures, and n/total (%) for categorical measures.

Table S3: Baseline characteristics of patients enrolled in Kolkata

	7 days PQ N=83	14 days PQ N=81
Age (years)	37.0 (25.0-48.0)	35.0 (30.0-46.0)
Sex		
Male	63/83 (75.9%)	66/81 (81.5%)
Female	20/83 (24.1%)	15/81 (19.5%)
Weight (kg)	60.0 (51.0-68.0)	58.8 (51.0-71.0)
Parasite density day -1 (/uL)	3253.5 (1107.4-7971.3)	3764.9 (1943.3-10339.1)
Body temperature day 0 (°C)	36.2 (35.9-36.5)	36.1 (35.9-36.2)
Presence of fever in the previous 48h	81/83 (98%)	79/81 (98%)
Haemoglobin day 0 (g/dL)	12.3 (1.6)	12.4 (1.9)
G6PD activity (U/gHb)	13.1 (11.5-15.1)	12.6 (11.1-14.2)
Primaquine mg/kg daily dose	0.6 (0.5-0.6)	0.3 (0.2-0.3)
Reported missed primaquine dose	11/83 (13%)	30/81 (37%)

Data are presented as mean (SD) or median (IQR) for continuous measures, and n/total (%) for categorical measures.

Figure S1: Trial profile of Ahmedabad site

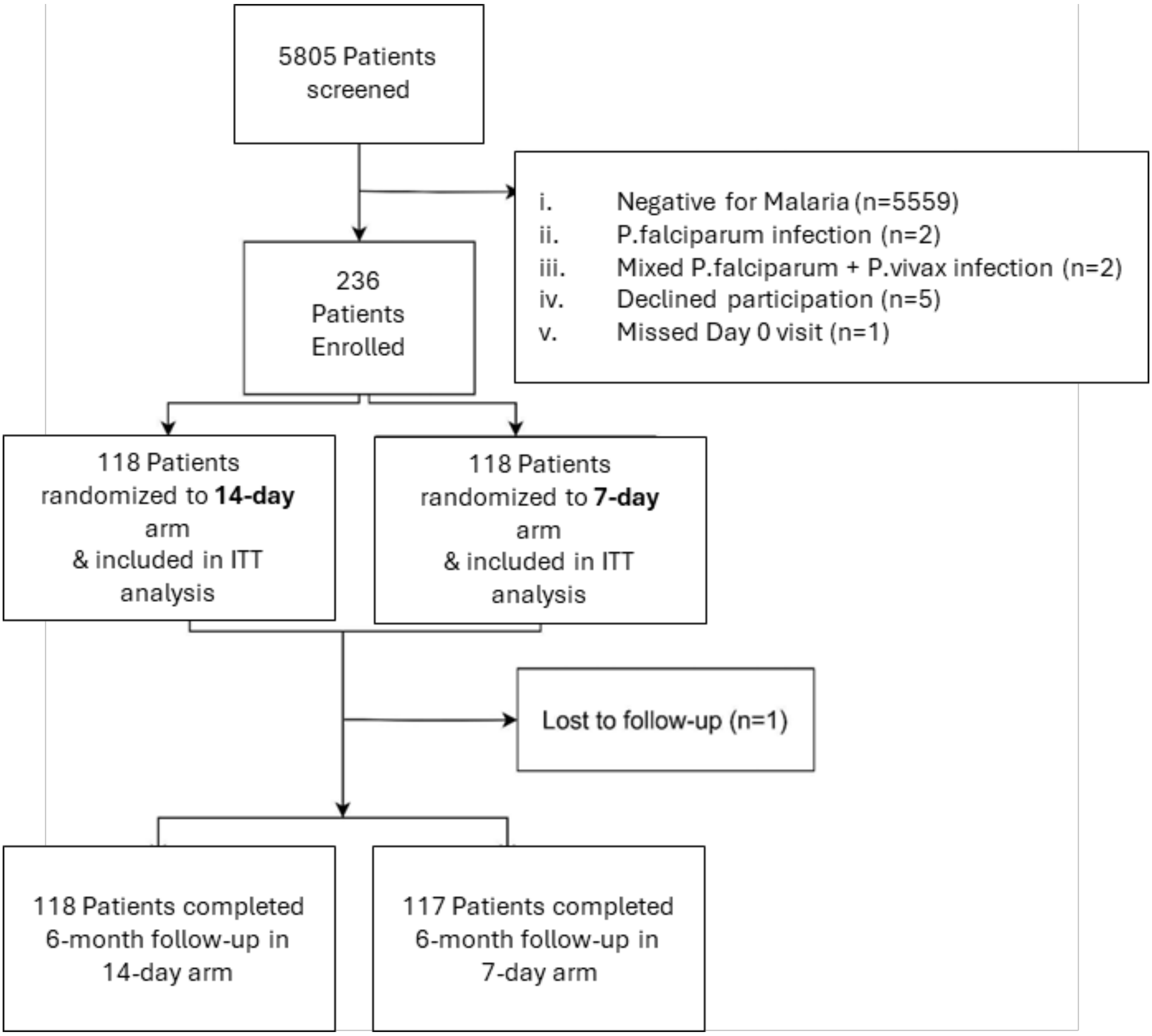

Figure S2: Trial profile of Kolkata site

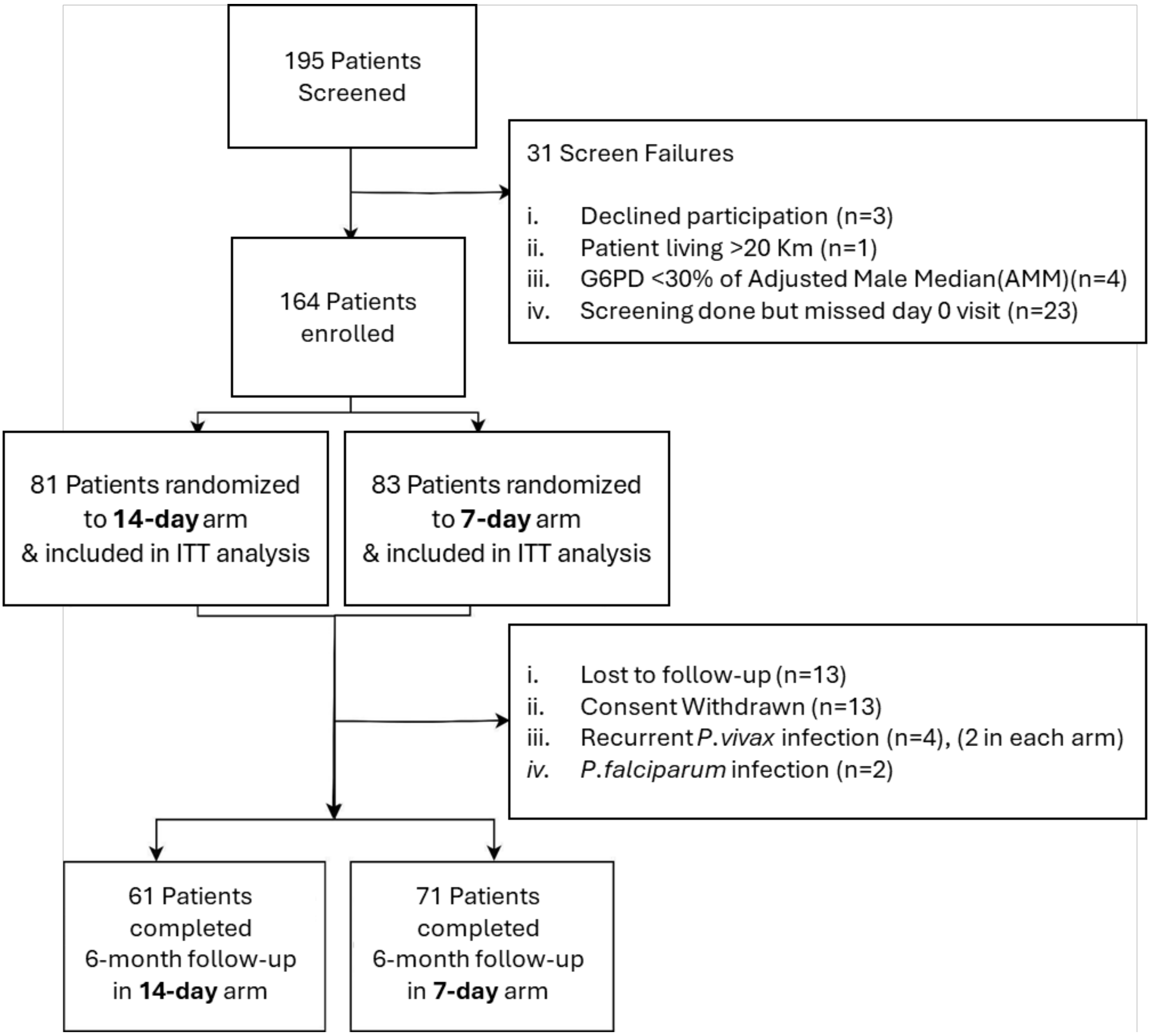

Figure S3: Primaquine mg/kg dose by arm

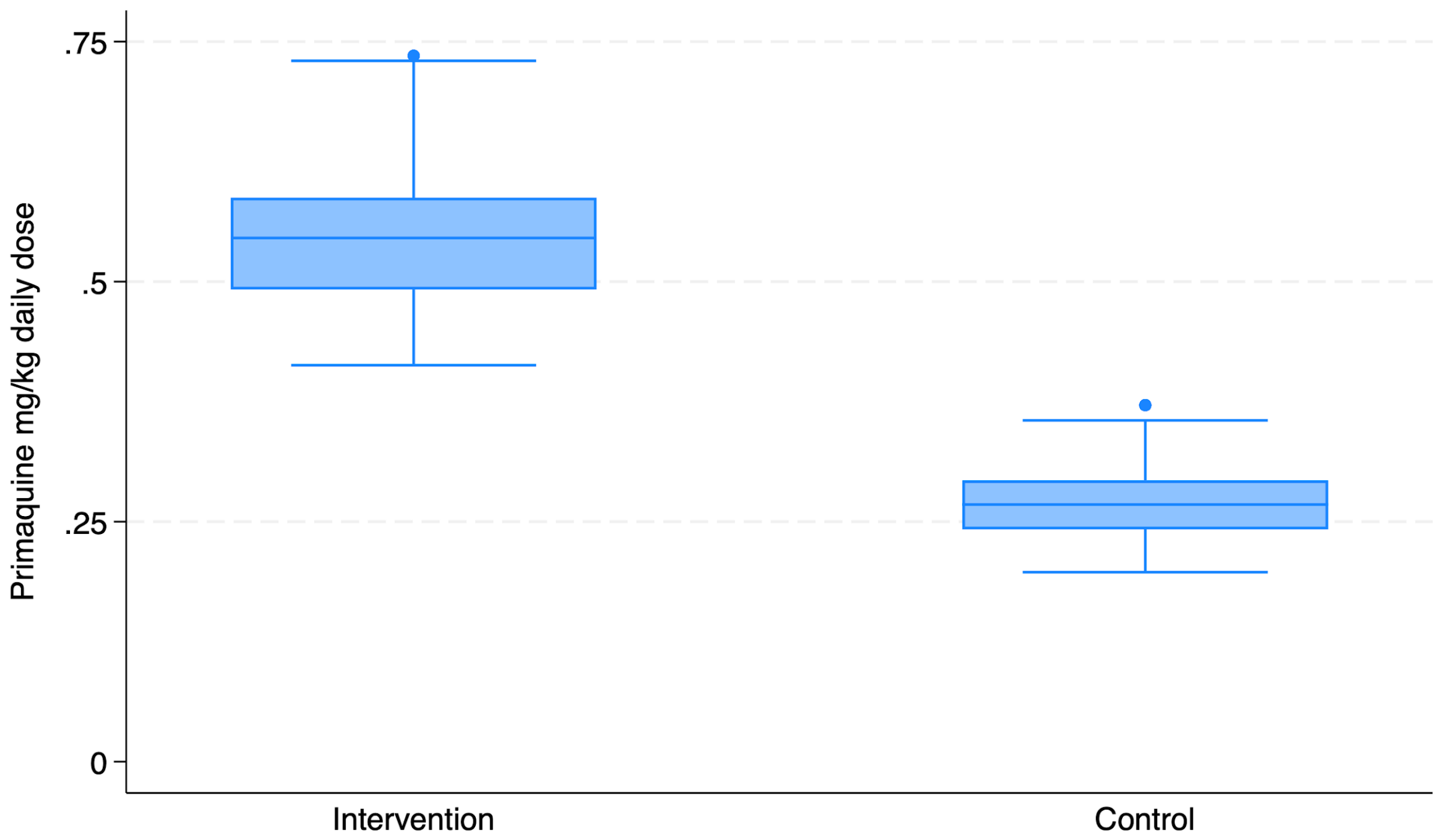

Intervention – 7 days 0.5 mg/kg/day primaquine, Control – 14 days 0.25 mg/kg/day primaquine

Figure S4: Cumulative incidence of any parasitaemia by day 180

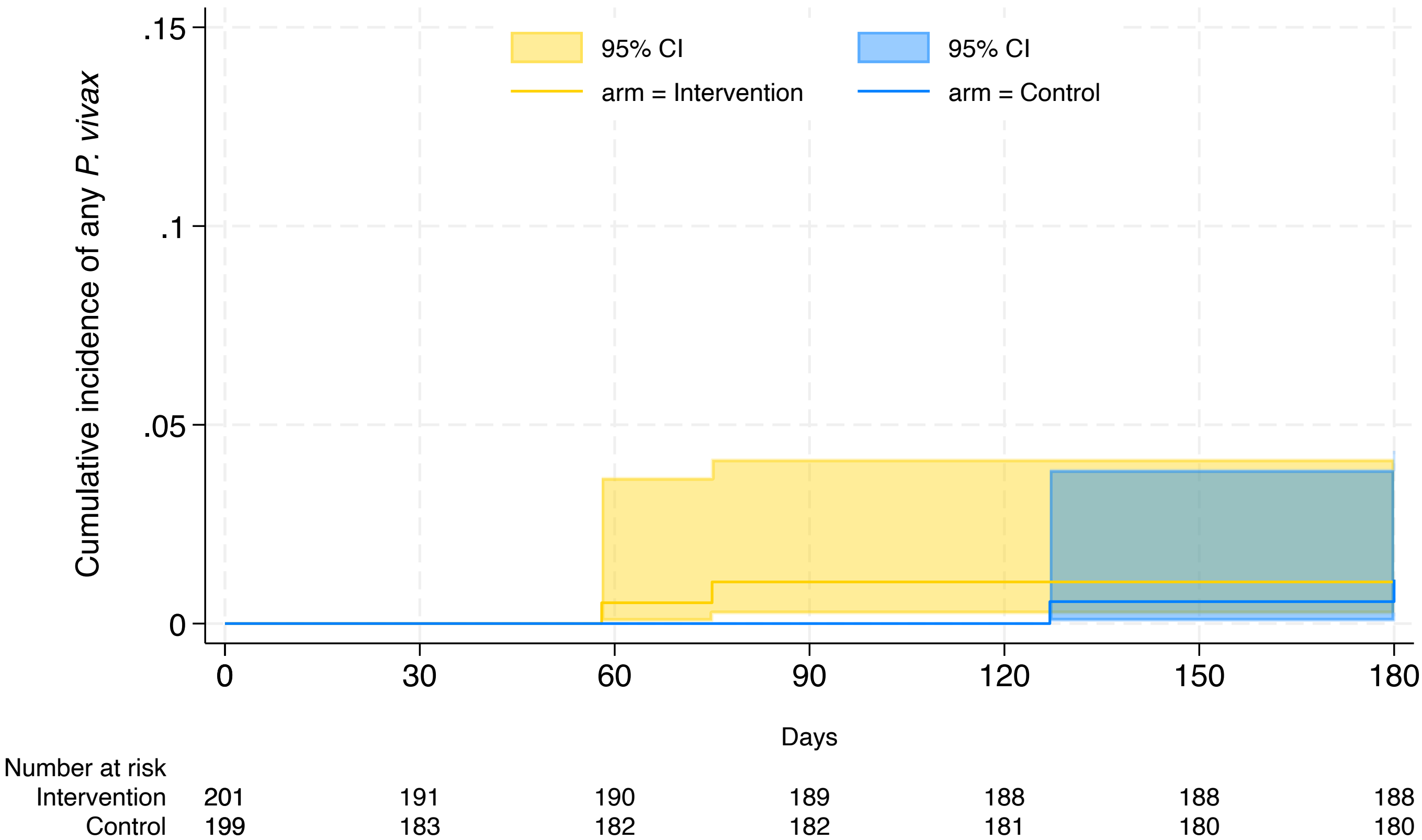

Intervention – 7 days 0.5 mg/kg/day primaquine, Control – 14 days 0.25 mg/kg/day primaquine

Figure S5: Cumulative incidence of symptomatic parasitaemia by day 180 at A) Ahmedabad and B) Kolkata

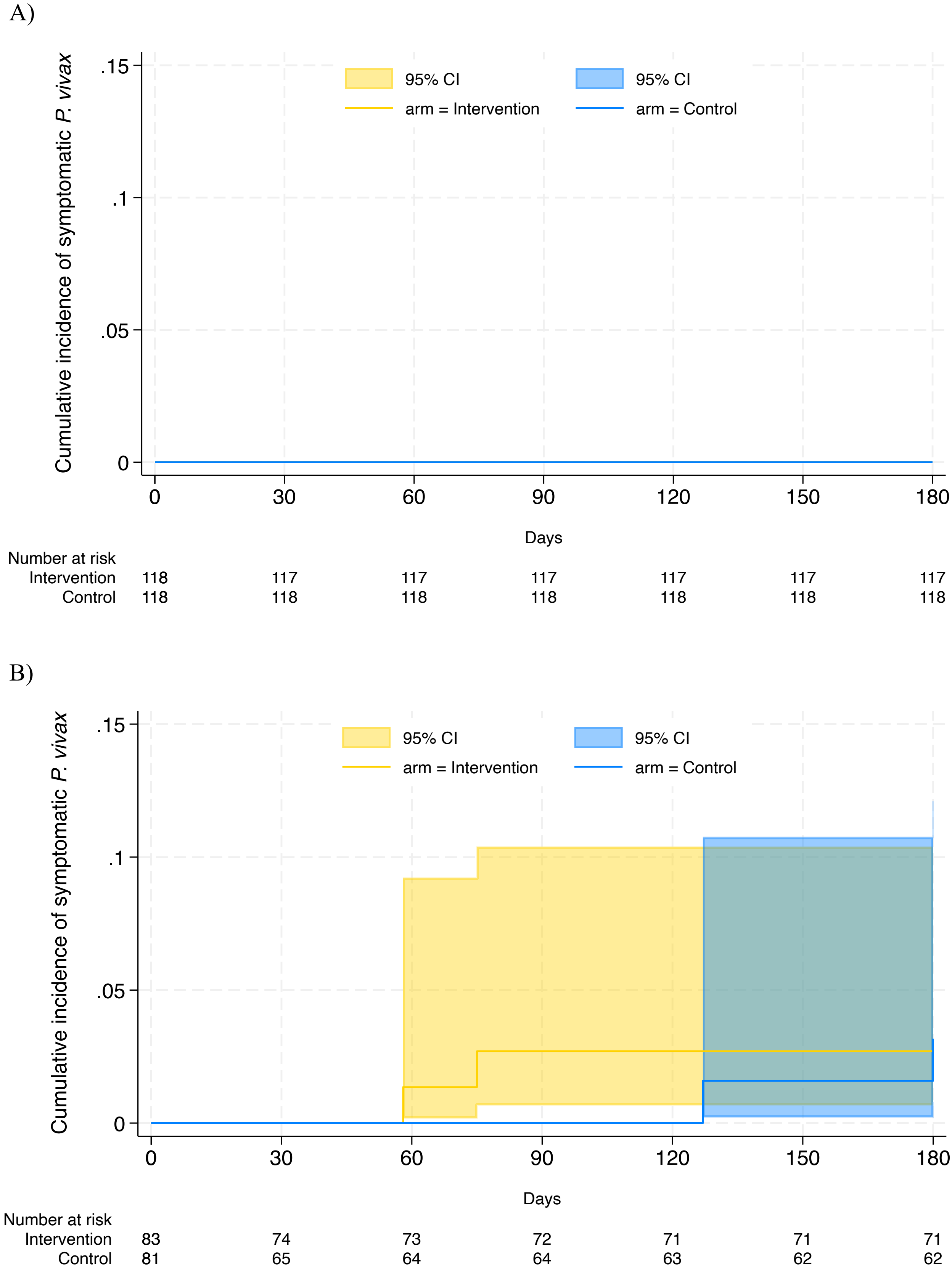

Intervention – 7 days 0.5 mg/kg/day primaquine, Control – 14 days 0.25 mg/kg/day primaquine

Figure S6: Cumulative incidence of symptomatic parasitaemia by day 180 if complete adherence to primaquine (per protocol analysis)

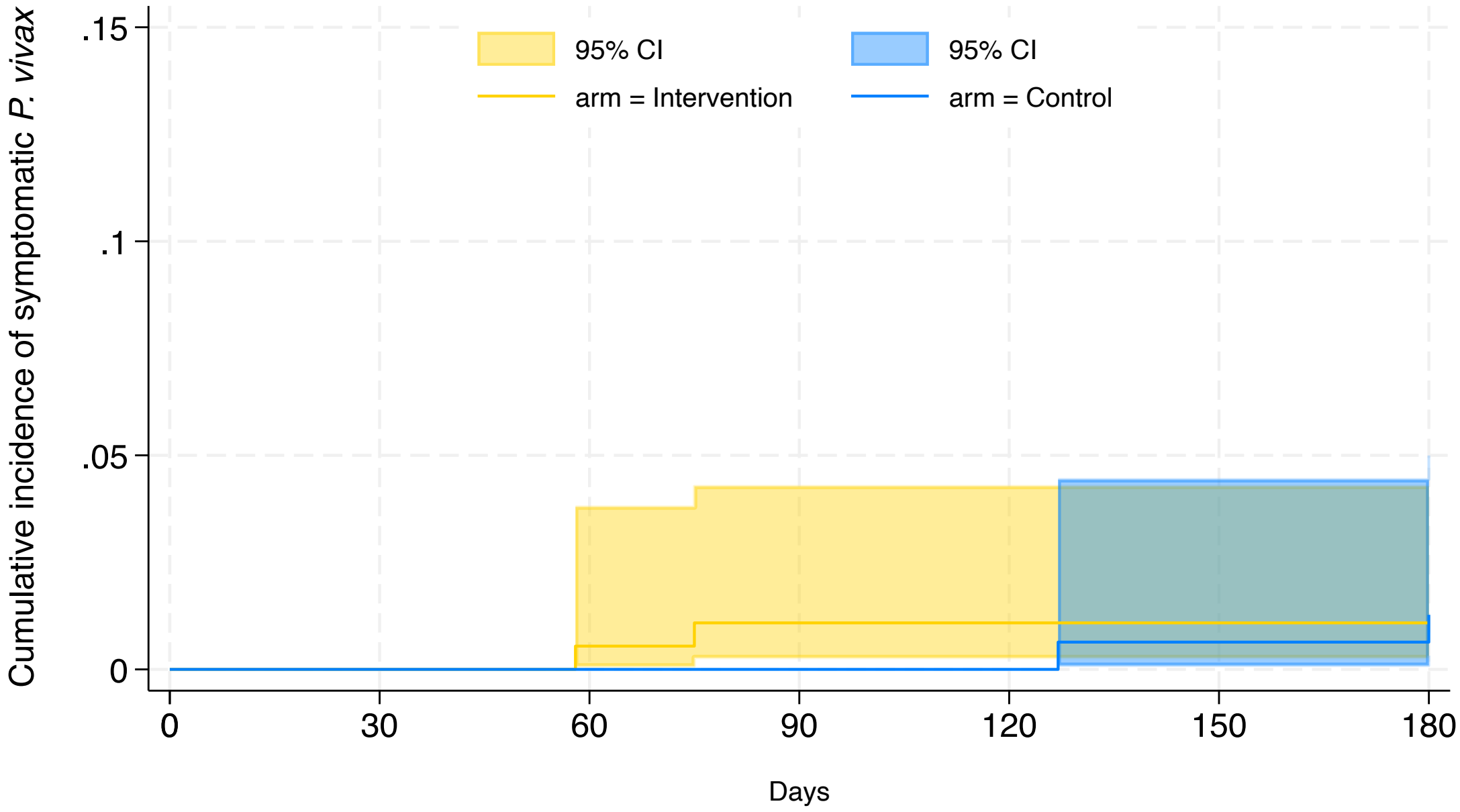

Number at risk							
Intervention	185	184	183	182	181	181	181
Control	159	159	158	158	157	156	156

Intervention – 7 days 0.5 mg/kg/day primaquine, Control – 14 days 0.25 mg/kg/day primaquine

Figure S7: Cumulative incidence of symptomatic parasitaemia by day 180 for A) males and B) females

A)

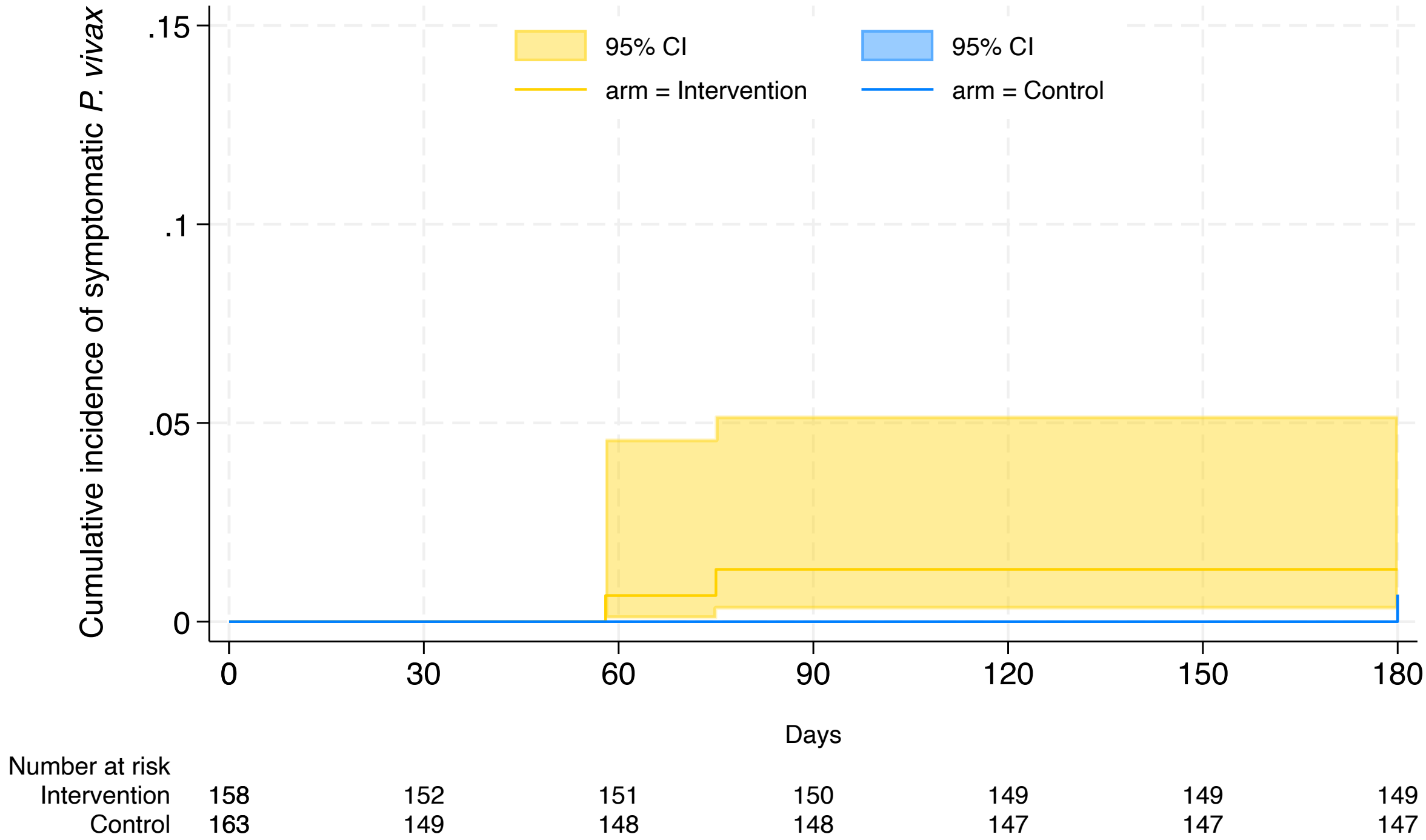

B)

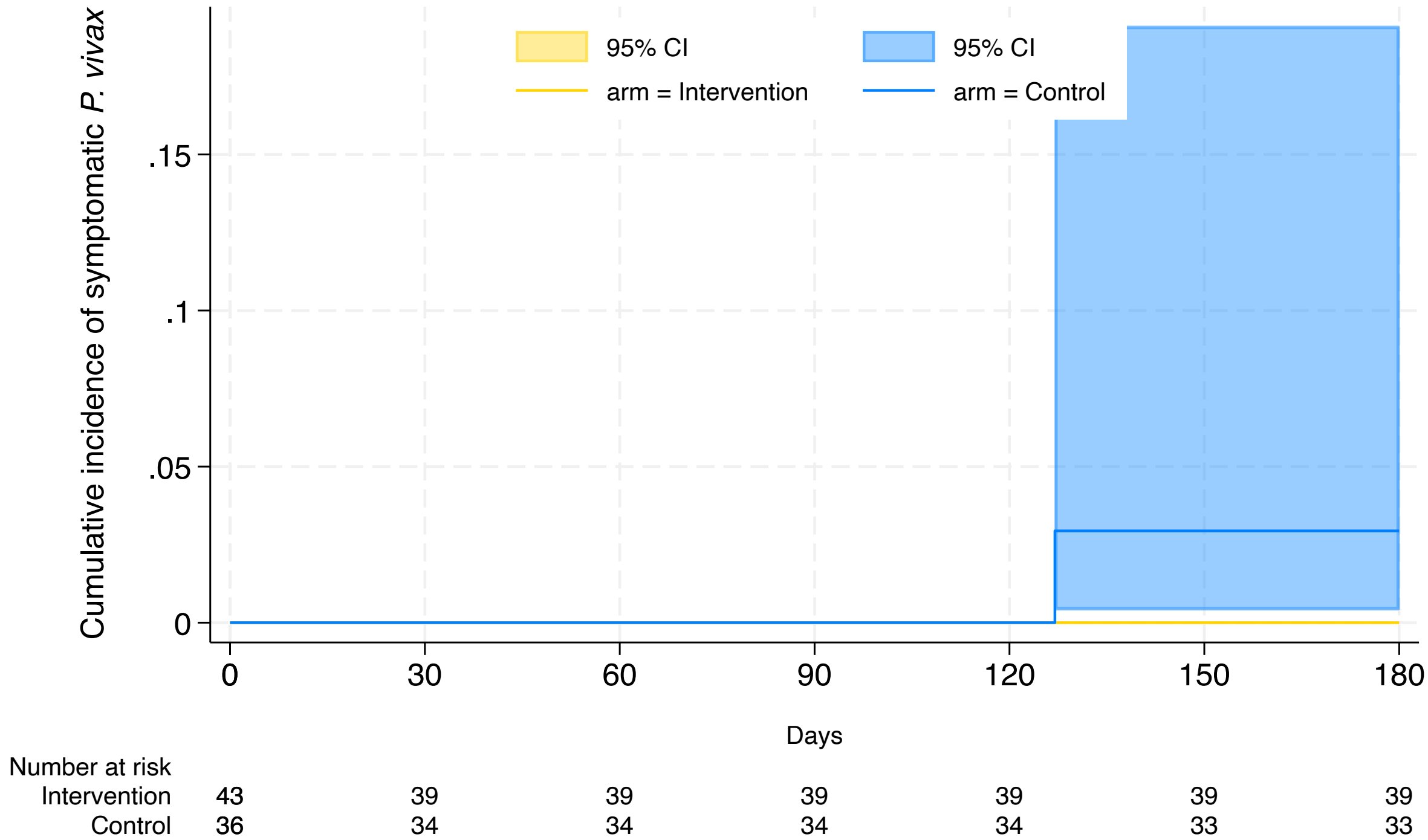

Intervention – 7 days 0.5 mg/kg/day primaquine, Control – 14 days 0.25 mg/kg/day primaquine
